# The Swiss Army Knife of Electrodes: Pillar[6]arene‐Modified Electrodes for Molecular Electrocatalysis Over a Wide pH Range

**DOI:** 10.1002/anie.202413144

**Published:** 2024-10-28

**Authors:** Helena Roithmeyer, Jan Bühler, Olivier Blacque, Isik Tuncay, Thomas Moehl, Cristiano Invernizzi, Florian Keller, Marcella Iannuzzi, S. David Tilley

**Affiliations:** ^1^ Department of Chemistry University of Zurich Winterthurerstrasse 190 8057 Zurich Switzerland; ^2^ Department of Science and High Technology Insubria University and INSTM 22100 Como Italy

**Keywords:** Host-Guest Chemistry, Electrocatalysis, pH Stability, Immobilized Catalysts, Pillararene

## Abstract

Molecularly‐modified electrode materials that maintain stability over a broad pH range are rare. Typically, each electrochemical transformation necessitates a specifically tuned system to achieve strong binding and high activity of the catalyst. Here, we report the functionalisation of *mesoporous* indium tin oxide (mITO) electrodes with the macrocyclic host molecule pillar[6]arene (PA[6]). These electrodes are stable within the pH range of 2.4–10.8 and can be equipped with electrochemically active ruthenium complexes through host–guest interactions to perform various oxidation reactions. Benzyl alcohol oxidation serves as a model reaction in acidic media, while ammonia oxidation is conducted to assess the systems performance under basic conditions. PA[6]‐modified electrodes demonstrate catalytic activity for both reactions when complexed to different guest molecules and can be reused by reabsorption of the catalyst after its degradation. Furthermore, the system can be employed to perform subsequent reactions in electrolyte with varying pH, enabling the same electrode to be utilised in multiple different electrocatalytic reactions.

## Graphical abstract

Pillar[6]arene‐functionalised indium tin oxide electrodes can be modified with electrocatalytically active ruthenium complexes through host–guest interactions. These versatile electrodes exhibit activity for reactions across a broad pH range (2.4–10.8) and can be reused after reabsorption of the desorbed catalyst. Different electrocatalytic transformations can be conducted under varying conditions utilising the same electrode.

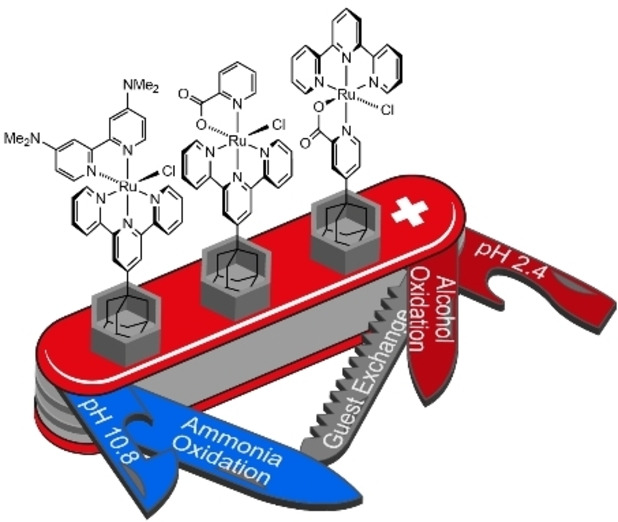

## Introduction

In the pursuit of a more sustainable and climate‐friendly future, there has been a notable boost in interest surrounding the application of electrochemistry.[^1^, ^2^] Owing to its heightened levels of control in terms of reaction rate and product selectivity over other approaches, electrocatalysis has carved out a distinct niche in the space of catalysis.[^3^, ^4^] Progress in sustainable electrocatalysis is inherently based on the development of efficient, non‐noble metal‐based electrode materials to not only diminish environmental impact but also increase their economic viability.^[5]^ Given the significant influence of electrode materials on the electron transfer rate and selectivity of a reaction, maximising their potential range and stability while minimising costs is paramount.^[6]^ Moreover, facile recyclability and reusability additionally favour the appeal of electrode materials. Rationalised design of such materials is essential for enhancing the aforementioned attributes and facilitating industrial‐scale implementation.[^7^, ^8^] One approach to address several of these challenges is the immobilisation of molecular electrocatalysts. The combination of intrinsic advantages of molecular species–such as high activity and selectivity–with the recyclability and reusability of solid‐state materials has the potential to yield efficient electrodes.[^9^, ^10^, ^11^]

Surface‐modification with macrocyclic molecules, such as cyclodextrins,^[12]^ calixarenes^[13]^ or pillararenes,^[14]^ offers a method for binding molecules through host–guest interactions. Such assemblies allow for the surface anchoring of molecular catalysts equipped with suitable binding units, such as naphthalene or adamantane groups.^[15]^ The anchoring of these catalysts through hydrophobic interactions enables their utilisation in electrochemical catalysis while maintaining their ability to be replaced with fresh catalyst molecules upon degradation.^[16]^ Surface‐bound metal complexes have been shown to retain the high specific activity and selectivity of molecular species while also benefiting from inherent advantages of heterogeneous electrocatalysts, including increased stability and reduced catalyst loading requirements.[^17^, ^18^, ^19^] Furthermore, immobilised catalysts allow for reactions to be conducted in aqueous media, even when the catalyst itself is hydrophobic. This eliminates certain constraints in catalyst design, particularly by enabling the ligand sphere of a metal complex to be tuned to a specific reaction without solubility restrictions. Importantly, the dynamic equilibrium of host–guest complex formation, as observed in solution, is shifted significantly in favour of the host–guest complexes when the host is bound to a surface, which results in a more stable binding than expected from homogeneous experiments.[^15^, ^16^]

Pillararenes show remarkable self‐assembly characteristics in both solution and on surfaces, directly dependent on their ring size and level of functionalisation.^[20]^ Huang and co‐workers pioneered surface‐modification with pillararenes when investigating functionalised gold particles for their host–guest complexation behaviours as well as their ability to function as reaction cavities.[^21^, ^22^] In a subsequent development, Inagi and co‐workers demonstrated the electrochemically induced oxidative generation of micron‐sized hexagonal cylinders from pillar[6]arene (PA[6]) on indium tin oxide (ITO) electrodes.^[23]^ PA[6] are highly symmetric, hexagonally shaped macrocycles composed of six methylene‐bridged 1,4‐diphenol units, with the bridging units *para* to each other.^[14]^ With a cavity size of approximately 6.7 Å, they accommodate suitable guest species through hydrophobic, van der Waals, CH‐π or π–π interactions. Strong binding is achieved with hydrocarbons, aromatic systems and cationic species.[^14^, ^24^, ^25^]. Pillararene assemblies have since been explored across various domains, including but not limited to medicinal,^[26]^ sensing^[27]^ and catalysis^[28]^ applications, leveraging their ability to selectively uptake guest molecules based on their distinctive properties regarding size and degree of functionalisation.[^29^, ^30^, ^31^] However, the functionalisation and adjustment of binding properties remains intricate, relying on sophisticated synthesis strategies.[^32^, ^33^]

Here, we report the self‐assembly of non‐functionalised pillar[6]arenes on a mesoporous indium tin oxide (mITO) surface, serving as the working electrode for different electrocatalytically driven reactions involving three catalytically active ruthenium complexes as the guest molecules. The versatility, pH stability and reusability of such electrodes is demonstrated in the electrochemical oxidation of benzyl alcohol (pH 1–2.4) and ammonia (pH 10.8–11.3).

## Results and Discussion

**Figure 1:**
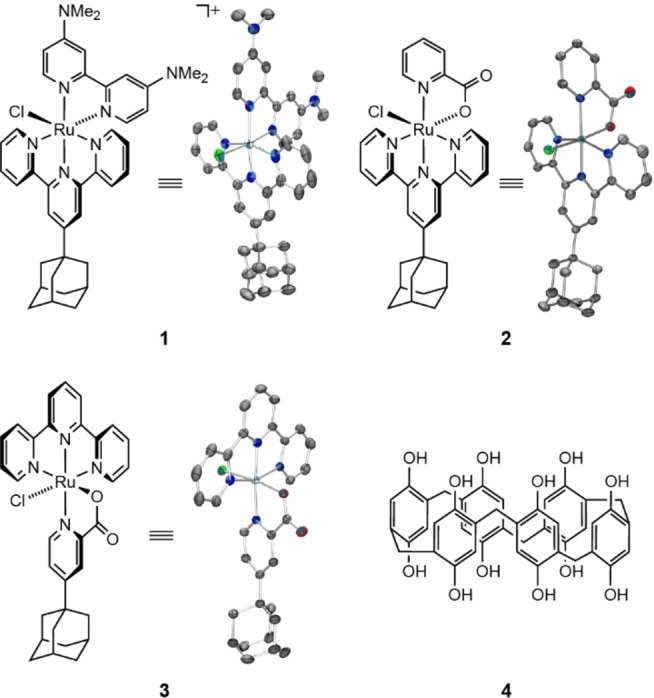
Chemical structures and ellipsoid displacement plots^[38]^ of [Ru(tpada)(bpy‐NMe_2_)(Cl)](PF_6_) (**1**),^[15]^ [Ru(tpada)(pic)(Cl)] (**2**) and [Ru(terpy)(ada‐pic)(Cl)] (**3**).^[39]^ Ellipsoids represent a 50 % probability; solvent molecules, counterions and hydrogen atoms are omitted for clarity. Chemical structure of pillar[6]arene‐OH (**4**).

The designs of the catalytically active ruthenium complexes (Figure 1) drew inspiration from our prior work on alcohol and ammonia oxidation with immobilised catalysts. [Ru(tpada)(bpy‐NMe_2_)(Cl)](PF_6_) (**1**, where tpada is 4’‐(adamantan‐1‐yl)‐2,2’ : 6’,2’’‐terpyridine and bpy‐NMe_2_ is 4,4’‐bis(dimethylamino)‐2,2’‐bipyridyl) has previously demonstrated activity in ammonia oxidation when tethered to a β‐cyclodextrin‐modified electrode.^[15]^ In contrast, [Ru(tpada)(pic)(Cl)] (**2**, where pic is picolinate) and [Ru(terpy)(ada‐pic)(Cl)] (**3**, where terpy is 2,2’ : 6’,2’’‐terpyridine and ada‐pic is 4‐(1‐adamantyl) picolinate) are adapted versions of an immobilised C−H activation catalyst.^[34]^ A common characteristic among these complexes is the presence of an adamantyl group, crucial for the stable binding to macrocyclic host molecules such as pillar[6]arene (PA[6], **4**). PA[6] was derived from 1,4‐bis(ethoxy)pillar[6]arene (PA[6]Et), synthesised with slight modifications to a literature procedure (see SI),^[35]^ followed by deprotection of the ether groups with boron tribromide. Immobilisation of **4** was achieved by simply immersing *mesoporous* indium tin oxide (mITO) electrodes in a 0.1 mM methanolic solution of the macrocycle for 90 min. Multilayer formation, observed during the soaking treatment, was eliminated by cyclic voltammetry (CV) in 0.2 M NaClO_4_ prior to functionalisation with ruthenium catalysts (Figure S1). The disappearance of the oxidative wave, attributed to the oxidation of the phenolic unit to hydroquinone, and the determined surface loading of **4** (1.81±0.17 nmol cm^−2^ geometric area), quantified through desorption studies in 1 M methanolic KOH (Figure S2, Table S1), signifies the removal of all non‐covalently bound PA[6]. When bound to the surface, PA[6] does not undergo oxidation to the hydroquinone under working conditions (Figure S3).

Theoretical calculations indicate that PA[6] initially pre‐organises with the phenol units oriented perpendicularly to the ITO surface through hydrogen bonding with an adsorption energy of −1.18 eV (Figure S4). This positions the cavity pointing away from the surface, allowing guest binding to occur. Following the initial hydrogen bonding, it is expected that dehydration occurs on the surface, leading to the formation of covalent bonds (Figure S5), similar to processes observed in the adsorption of carboxylic and phosphonic acids on TiO_2_.[^36^, ^37^] Following the dehydration, the interaction energy is −6.92 eV.

The anchoring of the three guest molecules (**1**–**3**) was accomplished by immersing PA[6]‐functionalised mITO electrodes in methanolic solutions of the complexes (0.1 mM) for 16 h. The geometric surface loading, determined by integration of the Ru^II^/Ru^III^ oxidative wave observed during CV measurements, was 2.05±0.10 nmol cm^−2^ for the three guest molecules (Table S2), which is within the error range for a 1 : 1 host–guest complex with PA[6]. We note that physisorbed Ru complexes do not stably bind to bare mITO, as evidenced by the negligible Ru^II^/Ru^III^ oxidative wave during CV measurements on the second scan (Figure S6). Guest molecules can be partially removed from the host cavity by immersing the functionalised electrodes in dimethyl sulfoxide for 90 min (Figure S7), which allows for the exchange of different guest molecules. Scan rate dependence experiments revealed a linear relationship of the peak current with respect to the scan rate, as expected for surface‐bound molecules (Figure S8).^[40]^

To assess the stability of the host–guest binding between PA[6] and compounds **1**, **2** and **3**, NMR titration studies were conducted in MeOD. High binding constants were observed for all three guest molecules in solution. Guests **1** and **2** exhibited K_11_ values of 2891±391 M^−1^ and 2260±362 M^−1^, respectively (Figures S9–10, Tables S3–4).

Notably, extreme peak broadening of the host‐specific signals was induced by guest **3**, attributed to an enhanced t_2_ relaxation time caused by restricted molecular movement of the host molecules resulting from strong binding (Figure S11, Table S5). Hence, the shift change in the methylene signal of PA[6] could not be precisely monitored, leading to a binding constant with a relatively large uncertainty (K_11_=16492±11860 M^−1^). To confirm this assumption on the enhancement of the t_2_ relaxation time, temperature‐dependent studies were conducted. Indeed, upon heating of the sample (308–323 K), signal sharpness was regained due to increased molecular movement (Figure S12).

**Figure 2:**
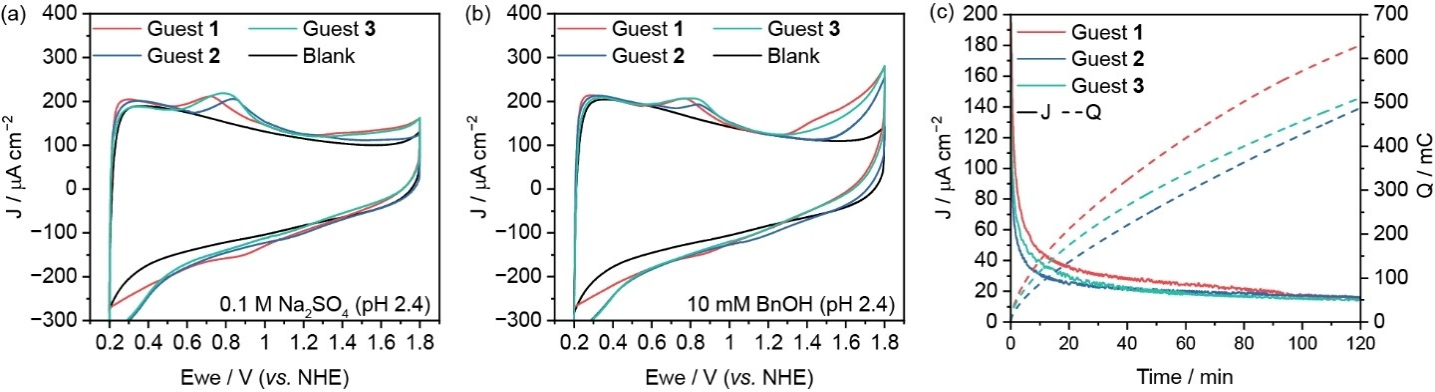
Cyclic voltammograms of (a) [Ru(tpada)(bpy‐NMe_2_)(Cl)](PF_6_) (Guest **1**, red), [Ru(tpada)(pic)(Cl)] (Guest **2**, blue), and [Ru(terpy)(ada‐pic)(Cl)] (Guest **3**, green) immobilised on a PA[6]‐modified mITO electrode in aqueous Na_2_SO_4_ (0.1 M, pH 2.4) and (b) after addition of benzyl alcohol (10 mM, 0.1 M Na_2_SO_4_, pH 2.4) at 100 mV s^−1^ (2^nd^ scan). (c) Representative plots of current densities and charge accumulation during chronoamperometry experiments with benzyl alcohol (10 mM) at 1.7 V vs. NHE.

Cyclic voltammetry experiments were carried out on the three host–guest complexes to gain insight into their electrochemical characteristics (Figure 2a). Given that the first electrocatalytic reaction under investigation was the oxidation of benzyl alcohol to benzaldehyde, a reaction that proceeds efficiently under acidic conditions, the experiments were conducted in aqueous electrolyte at pH 2.4. Under these conditions, the chloride ligand of the guest molecules is rapidly exchanged, forming the corresponding solvato complex (Figure S13).[^15^, ^34^] The three catalysts showed the expected redox waves for the Ru^II^/Ru^III^ oxidation at 0.7, 0.8 and 0.75 V vs. NHE for guests **1** (red), **2** (blue) and **3** (green), respectively. A less pronounced wave was present for the Ru^III^/Ru^IV^ oxidation between 1.35–1.45 V vs. NHE for all Ru complexes. For compound **1**, the pH dependence of the redox potentials was investigated by construction of a Pourbaix diagram in the pH range of 1.0–12.0 (Figure S14). The Ru^II^/Ru^III^ redox couple displays pH independence in the ranges of 1.0–3.0 and 9.0–12.0. A slope of −69 mV pH^−1^ indicates approximately Nernstian behaviour and the presence of a proton‐coupled electron transfer (PCET) between pH 3.0–9.0. For the Ru^III^/Ru^IV^ couple, the transfer of an electron is coupled to the transfer of a single proton in the range of 1.0–3.0 and two protons between 3.0–12.0. We note that there is no indication that the *N,N*‐dimethylamine substituents are protonated under these conditions. Upon the addition of benzyl alcohol substrate (10 mM), catalytic onset is observed at 1.3 V vs. NHE for **1** and **3**, roughly aligning with the aforementioned Ru^III^/Ru^IV^ oxidative wave (Figure 2b). A larger overpotential was required to initiate alcohol oxidation with complex **2**, where catalytic onset starts at 1.45 V vs. NHE (Figure 2b). Cycling experiments to test the stability of the host–guest complexes at pH 2.4 were performed with guest **1** before and after the addition of the substrate (Figure S15a and S15b). Based on these results, chronoamperometry (CA) experiments were conducted to evaluate the catalytic activity of the Ru catalysts. Electrocatalysis was performed at 1.7 V vs. NHE for a duration of 2 h (Figure 2c). Product formation and faradaic efficiency (FE) were quantified by ^1^H NMR spectroscopy of the resultant reaction mixtures (Figure S16). The outcomes of these catalytic assays are summarised in Table 1. Notably, the faradaic efficiency of all host–guest complexes remained consistently high, ranging between 88–92 %, indicating minimal background water oxidation. This observation was further confirmed when chronoamperometry was performed in the absence of the substrate (Table S6). Turnover numbers (TON) were in the low hundreds, with the best‐performing catalyst (**1**) achieving 432±36 for the initial absorption. We note that samples prepared by physisorption of the Ru complexes on mITO electrodes (bare mITO, without PA[6]) did not yield significant charge accumulation or product formation (Table S6). Due to the small quantity of catalyst relative to the amount of substrate present in the electrolyte, the overall conversion remains low. However, we have previously shown that by decreasing the ratio of electrolyte volume to surface area, a near quantitative conversion of the starting material can be achieved.^[19]^

**Table 1:** Results of oxidation reactions with benzyl alcohol.

Catalyst	Absorption	Q [mC]	NMR Yield [mM]	FE [%]	TON
Guest 1	1^st^	568±59	0.34±0.03	92±2	432±36
Guest 1	2^nd^	615±28	0.35±0.02	88±2	449±25
Guest 2	1^st^	451±29	0.26±0.01	88±5	343±6
Guest 2	2^nd^	522±59	0.31±0.03	92±2	416±43
Guest 3	1^st^	467±34	0.27±0.02	90±1	347±25
Guest 3	2^nd^	486±17	0.29±0.01	92±1	369±16

Throughout the CA experiments, a gradual degradation of the catalyst became evident, manifested by the diminishing current densities over time. This degradation can arise from various mechanisms, including *N*‐oxidation of the pyridyl units, oxidative cleavage of the anchoring unit or ligand dissociation.[^41^, ^42^] Nonetheless, PA[6] remained stably anchored onto the mITO electrode. This was evidenced by reabsorption of the catalyst upon immersion of the used electrodes into a fresh solution of the Ru complexes (Table S7). Following the reabsorption of the catalysts, a subsequent round of catalysis can be performed, yielding similar results to the first absorption (Table 1, 2^nd^ Absorption and Table S7).

FE and TON's were in the same range as the initial absorption, for the three systems. In an effort to push the host–guest‐modified electrodes to their limits, electrocatalysis was conducted at pH 1 (0.1 M H_2_SO_4_) and pH 0 (1 M H_2_SO_4_). Experiments at pH 1 demonstrated successful catalysis with reduced product formation compared to previous examples at pH 2.4 (Table S8). Moreover, it became evident that the binding of PA[6] to mITO was less stable at this pH, as indicated by decreased catalytic performance for the 2^nd^ absorption, reaching only approximately 80 % of the initial results. At pH 0, PA[6] was desorbed from the electrode almost instantaneously, with no observable catalytic current.

**Figure 3:**
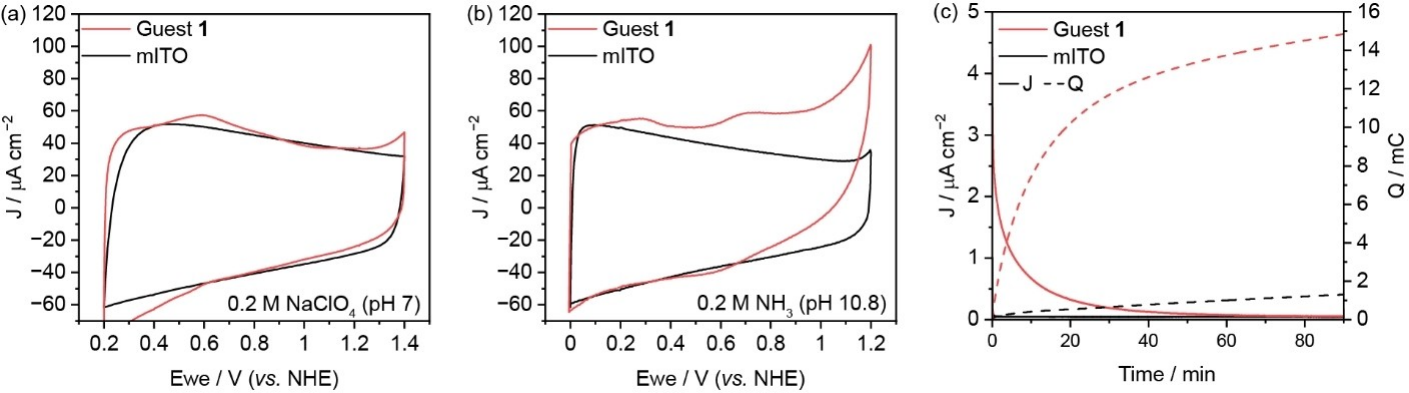
Cyclic voltammograms of [Ru(tpada)(bpy‐NMe_2_)(Cl)](PF_6_) (Guest **1**, red) immobilised on a PA[6]‐modified mITO electrode in (a) aqueous NaClO_4_ (0.2 M, pH 7, 20 mV s^−1^, 2^nd^ scan) and (b) aqueous NH_3_ (0.2 M, phosphate buffer at pH 10.8, 20 mV s^−1^, 2nd scan). (c) Representative plots of current densities and charge accumulation during chronoamperometry experiments with aqueous NH_3_ (0.2 M, pH 10.8) at 0.9 V vs. NHE.

To evaluate the stability and catalytic performance of electrodes modified with a PA[6] host–guest complex for reactions under alkaline conditions, ammonia oxidation experiments were conducted. CV traces of anchored guest **1** and bare mITO were recorded in neutral aqueous NaClO_4_ electrolyte, revealing a broad redox feature at around 0.7 V vs. NHE for the oxidation of the Ru core in **1** (Figure 3a, red) compared to the bare electrode (black). In the presence of ammonia (0.2 M, phosphate buffer at pH 10.8), catalytic onset is evident at approximately 0.6 V vs. NHE, coinciding with the Ru^III^/Ru^IV^ oxidation (Figure 3b). Ru^IV^ has been previously identified as the active species for ammonia oxidation in aqueous media.^[43]^ Detailed mechanistic insights for the direct ammonia to nitrate oxidation have been given by Chen et al.^[44]^ and Brudvig et al.^[45]^ Furthermore, a weak Ru^II^/Ru^III^ transition can be observed at approximately 0.25 V vs. NHE for **1** (Figure 3b). We note that little catalytic activity is observed for CA measurements at 0.5 V vs. NHE (Ru^III^) with the amount of charge passed similar to blank mITO (3 mC). Ammonia oxidation was also observed on bare mITO, albeit with an increased overpotential. Cycling experiments with guest **1** were performed before and after the addition of ammonia to determine the PA[6] and catalyst stability under these conditions (Figure S15c and S15d). Electrocatalysis was carried out for 90 min at 0.9 V vs. NHE (Ru^IV^), a potential where background oxidation by the electrode material is minimal (Figure 3b and c, black line). Nitrate formation was identified as the main product with FE of up to 100±4 %, as determined by the Griess test[^15^, ^46^] (Figure S17, Table S9–10). Subsequent reabsorption of the guest molecules demonstrated decent stability of the surface‐bound PA[6]. However, based on the charge accumulation during the experiment, a decrease in catalytic activity of approximately 30–40 % was observed (Figure S18). This suggests the presence of fewer host–guest complexes, indicating partial desorption of PA[6]. With limited nitrate production in these experiments, product quantification by the colourimetric Griess test became impossible due to the concentration falling below the detection limit. Nevertheless, electrocatalysis could be repeated multiple times utilising the same PA[6]‐modified electrode (Figure S18). When the pH of the substrate solution was increased to 11.3 (0.2 M NH_3_, without supporting electrolyte), electrocatalysis remained possible with FE of up to 57 % for the conversion to nitrate. However, reabsorption was not achievable at this pH, as evidenced by the absence of redox peaks during CV measurements (Figure S19, red). This indicates that PA[6] was not stably bound at this pH. This could either be caused by the desorption of the host molecule or etching of the mITO surface in highly basic conditions. Measurements of the electrochemically active surface area (Figure S20) revealed a difference of roughly 10% before and after chronoamperometry at pH 10.8, whereas it remained largely unchanged at pH 2.4.

**Figure 4:**
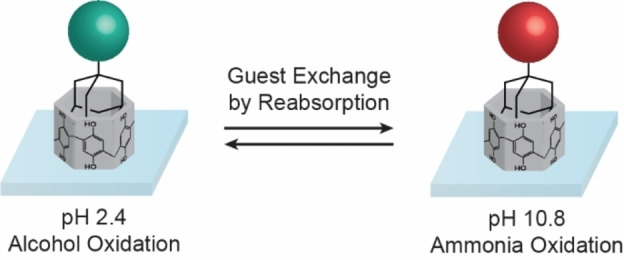
Schematic representation of multifunctional mITO electrodes modified with PA[6]. Electrodes can be used for alcohol oxidation followed by ammonia oxidation, and *vice versa*, by simply reabsorbing a different catalyst after use.

To demonstrate the versatility of PA[6]‐modified electrodes in recycling and repurposing for different reactions at varying pH levels, reabsorption experiments were conducted (Figure S21–22). PA[6]‐modified electrodes can be used sequentially for alcohol and ammonia oxidation in different aqueous environments (Figure 4). For this purpose, guest molecules can be exchanged with different catalysts to enable variable reactivities. When alcohol oxidation (guest **3**) is performed before ammonia oxidation (guest **1**), the surface loading of the reabsorption is equivalent to the initial absorption. However, when ammonia oxidation is conducted first, the surface loading of the reabsorption is lower due to a partial loss of PA[6] under alkaline conditions. Catalytic results are summarised in Table S11 and S12. This versatility can also be achieved by employing the same catalyst (guest **1**) for both reactions.

This marks an initial step in the development of an electrode capable of performing reactions over a broad pH range and for varying reactions. With the possibility to tailor guest molecules for specific reactions, PA[6]‐modified mITO electrodes hold promise as a foundation for the advancement of multiuse electrode systems.

## Conclusion

In summary, we have demonstrated the modification of *mesoporous* indium tin oxide electrodes with macrocyclic pillar[6]arene and subsequent binding of three ruthenium complexes via host–guest interactions for further utilisation in electrocatalysis. These host–guest‐modified electrodes were employed for alcohol oxidation (pH 2.4) as well as ammonia oxidation (pH 10.8), highlighting reasonable stability in both acidic and alkaline media. The catalysts were reabsorbed to undertake a second round of catalysis, indicating prolonged stability of the surface‐bound PA[6]. pH‐dependent experiments revealed that alcohol oxidation remains feasible down to pH 1, despite observing partial desorption of the host. Ammonia oxidation remains viable above pH 11, however, reabsorption is hindered due to the low stability of surface‐bound PA[6] within this pH range. Further functionalisation of the host, PA[6], could enhance the system stability and enable electrocatalysis beyond the observed pH range for unfunctionalised PA[6]. Beyond the reabsorption and repetitive electrocatalysis under identical conditions, we have further shown that PA[6]‐modified electrodes can be used for subsequent reactions in different media. This was evidenced by performing alcohol oxidation followed by ammonia oxidation (and *vice versa*) on the same sample, achieved simply by reabsorbing the same or a different catalyst. This represents a first step towards the production of pH‐stable host–guest‐modified electrodes for electrocatalysis.

## Supporting Information

The authors have cited additional references within the Supporting Information.[^47^, ^48^, ^49^, ^50^, ^51^, ^52^, ^53^, ^54^, ^55^, ^56^, ^57^, ^58^, ^59^, ^60^, ^61^, ^62^, ^63^, ^64^]

## Conflict of Interests

The authors declare no conflict of interest.

1

## Supporting information

As a service to our authors and readers, this journal provides supporting information supplied by the authors. Such materials are peer reviewed and may be re‐organized for online delivery, but are not copy‐edited or typeset. Technical support issues arising from supporting information (other than missing files) should be addressed to the authors.

Supporting Information

## Data Availability

The data that support the findings of this study are available from the corresponding author upon reasonable request.
