# Supplementary material for: The Swiss Army Knife of Electrodes: Pillar[6]arene‐Modified Electrodes for Molecular Electrocatalysis Over a Wide pH Range
Source: Angew Chem Int Ed Engl. 2024 Oct 28;64(1):e202413144. doi: 10.1002/anie.202413144 (PMC11701350; doi:10.1002/anie.202413144)
Supplement: Supplementary file 1 — Supporting Information [file ANIE-64-e202413144-s001.pdf]

Angewandte Chemie
Eine Zeitschrift der Gesellschaft Deutscher Chemiker

Supporting Information

The Swiss Army Knife of Electrodes: Pillar[6]arene-Modified Electrodes for Molecular Electrocatalysis Over a Wide pH Range

H. Roithmeyer, J. Bühler, O. Blacque, I. Tuncay, T. Moehl, C. Invernizzi, F. Keller, M. Iannuzzi, S. D. Tilley**

Supporting Information

The Swiss Army Knife of Electrodes: Pillar[6]arene-Modified Electrodes for Molecular Electrocatalysis Over a Wide pH Range

Helena Roithmeyer,*[a]+ Jan Bühler,[a]+ Olivier Blacque,[a] Isik Tuncay,[a] Thomas Moehl,[a] Cristiano Invernizzi,[a][b] Florian Keller,[a] Marcella Iannuzzi,[a] S. David Tilley*[a]

[a] Dr. H. Roithmeyer, J. Bühler, Dr. O. Blacque, I. Tuncay, Dr. T. Moehl, C. Invernizzi, F. Keller, Prof. Dr. M. Iannuzzi, Prof. Dr. S. D. Tilley
Department of Chemistry
University of Zurich
Winterthurerstrasse190, 8057 Zurich, Switzerland
E-mail: helena.roithmeyer@chem.uzh.ch, david.tilley@chem.uzh.ch

[b] C. Invernizzi
Department of Science and High Technology
Insubria University and INSTM
22100 Como, Italy

+ H.R. and J.B: contributed equally to this study.

Table of Contents

General 3
NMR spectroscopy 3
Mass spectrometry 3
UV-Vis spectroscopy 3
X-ray crystallography 3
Electrochemistry 4
Experimental 4
Electrode preparation 4
Solution preparation 5
Electrochemical methods 5
Surface loading of PA[6] 6
Oxidative stability of the surface-bound pillar[6]arene 7
Theoretical calculations 8
Surface loading of the guest catalysts 10
Physisorption 11
Desorption of the guest 11
Scan rate dependence 12
NMR Host-Guest binding studies 13
NMR temperature studies 18
Ligand exchange 19
Pourbaix diagram of guest 1 19
pH Stability 20
Alcohol oxidation 21
Quantification of products after alcohol oxidation 21
Ammonia oxidation 23
Quantification of nitrate 23
Guest, pH and medium exchange 27
Synthesis and Characterisation 30
References 47

General

All reagents and solvents were purchased from Merck/Sigma Aldrich or Chemie Brunschwig. Solvents were used reagent grade (99%), H_2O was used MilliQ grade. Fluorine-doped tin oxide (FTO) glass plates were ordered from Pilkington NSG TEC 15 (2.2mm, 12-15 Ω/ sq.) Indium tin oxide nanopowder particles (ITO. In_2O_3:SnO_2= 90:10, 99.99%, 18 nm) and proprietary nanopowder dispersant were purchased from *US Research Nanomaterials Inc.*, Houston, TX, USA.

Terpyridine adamantane (**1a**), [Ru$^{(III)}$(tpada)(Cl_3)] (**1b**) and [Ru(tpada)(bpy-NMe_2)(Cl)](PF_6) (**1**) were synthesised according to our previous work.[15] [Ru$^{(III)}$(terpy)(Cl_3)] and Ethyl-4-(1-adamantyl)picolinate (**3a**) were synthesised according to published procedures. [47,48] Pillar[6]arene (**4a**) was synthesised similarly to a literature-known procedure,[35] the exact synthesis can be found under synthesis and characterisation.

NMR spectroscopy

All spectra were acquired on a 400 or 500 MHz *Bruker* Avance spectrometer. All spectra were referenced according to their residual solvent signals[49] and processed with *Mnova*.

Mass spectrometry

High-resolution electrospray mass spectra (HR-ESI-MS) were recorded on a timsTOF Pro TIMS-QTOF-MS instrument (*Bruker Daltonics GmbH*, Bremen, Germany). The samples were dissolved in (e.g. MeOH) at a concentration of ca. 50 µg ml^{-1} and analysed via continuous flow injection (2 µL min^{-1}). The mass spectrometer was operated in the positive (or negative) electrospray ionisation mode at 4’000 V (-4’000 V) capillary voltage and -500 V (500 V) endplate offset with a N_2 nebuliser pressure of 0.4 bar and a dry gas flow of 4 L min^{-1} at 180°C. Mass spectra were acquired in a mass range from m/z 50 to 2’000 at ca. 20’000 resolution (m/z 622) and at 1.0 Hz rate. The mass analyser was calibrated between m/z 118 and 2’721 using an Agilent ESI-L low-concentration tuning mix solution (*Agilent*, USA) at a resolution of 20’000, giving a mass accuracy below 2 ppm. All solvents used were purchased in the best LC-MS quality.

UV-Vis spectroscopy

The measurements were conducted on a SHIMADZU UV-3600 Plus Spectrophotometer. The related solvent (*e.g.* H_2O or DMSO) was used as a baseline blank.

X-ray crystallography

Single crystal X-ray diffraction data were collected at 160.0(1) K on a *Rigaku OD Synergy/Hypix* diffractometer using the copper X-ray radiation (λ = 1.54184 Å) from a dual-wavelength X-ray source and an *Oxford Instruments Cryojet XL* cooler. The selected suitable single crystal was mounted using polybutene oil on a flexible loop fixed on a goniometer head and immediately transferred to the diffractometer. Pre-experiment, data collection, data reduction and analytical absorption correction[50] were performed with the program suite *CrysAlisPro*. [51] Using *Olex2*,[52] the structure was solved with the *SHELXT*[53] small molecule structure solution program and refined with the *SHELXL* program package[54] by full-matrix least-squares minimisation on F^2. *PLATON*[55] was used to check the result of the X-ray analysis.

Deposition Numbers 2352566 (for **2**) and 2352567 (for **3**) Contain the Supplementary Crystallographic Data for This Paper. These Data Are Provided Free of Charge by the Joint

Cambridge Crystallographic Data Centre and Fachinformationszentrum Karlsruhe Access Structures Service *via* www.ccdc.cam.ac.uk/structures.

Electrochemistry

Electrochemical measurements were conducted on a *BioLogic* SP-150 potentiostat using a three-electrode setup. FTO with spin-coated mITO (see experimental) and functionalised host (H) and guest (G) served as the working electrode. A platinum wire was used as the counter electrode. The reference electrode consisted of an Ag wire coated with AgCl in a 3 M KCl solution.

Cyclic voltammograms were recorded at 20, 50 or 100 mV s^{-1} (as stated), starting from the lowest potential and scanning in positive direction.

Alcohol Oxidation: Solvent volumes of 8 mL were used in chronoamperometry experiments, and solutions were stirred at 100 rpm with a magnetic stir bar.

Ammonia oxidation: 6 to 10 mL solvent volumes were used for chronoamperometry without stirring the solution.

Voltages are reported *vs*. the normal hydrogen electrode (NHE) by conversion of the measured potential according to the following equation: $E_{NHE} = E_{Ag/AgCl} + 0.199$ V.

If not stated otherwise the 2nd CV cycle is shown in all figures in the manuscript and the SI.

Electrochemical cell

An H-cell, with glass frits (P4 and P5) as membranes, was used for all electrochemical measurements. The anode solution ranges from 6 to 10 mL depending on the experiment and electrode size.

Experimental

Electrode preparation

Preparation of ITO spin-coating suspension and mITO-coated electrodes: 2 g of ITO particles (18 nm, 99.99%) were sonicated in 10 mL EtOH for 30 min before a solution of ethyl cellulose (200 mg), alcohol surfactant (proprietary nanopowder dispersant, 225 mg), and 5 g terpineol in 5 mL EtOH was added. The blue suspension was sonicated for 10 min, and the solvent was removed *in vacuo* to form a viscous blue paste. Afterwards, 3 mL of the paste were further diluted with 7 mL EtOH, sonicated for 10 min and used for spin-coating.[19]

Preparation of the mITO electrodes: The mITO electrodes were prepared as published in our previous work[15] by spin coating 3 × 180 µL nanoparticle suspension in EtOH onto FTO glass slides (geometric area between 3 cm^2 and 3.8 cm^2), which were previously sonicated in acetone, H_2O containing alkaline detergent (Deconex 11 Universal), and EtOH/ H_2O (7:3) for 10 min each. The FTO glass was partially covered with Kapton tape, and freshly sonicated ITO spin-coating suspension was spin-coated for 20 s (2000 rpm, 500 rpm s^{-1}) onto the plates. The solvent was evaporated on a hot plate at 120 °C for 10 min, and the spin coating step was repeated twice.

After the third cycle, the tape was removed, and the electrodes were heated on a hot plate at 450 °C for 60 min.

Solution preparation

Benzyl alcohol solution: A 0.1 M Na_2SO_4 solution was prepared by dissolving 7.10 g of Na_2SO_4 in 500 mL of H_2O. The pH of the solution was adjusted to 2.4 with drops of conc. H_2SO_4. To 10 mL of this solution,10.4 µL of benzyl alcohol (10 mM) was added.

Phosphate buffer: 8.14 g potassium phosphate dibasic and 444 mg potassium phosphate monobasic were dissolved and diluted to 500 mL with deionised H_2O to give a 0.1 M phosphate buffer with a pH of 8.00. [56]

Ammonia solution: A 0.2 M ammonia solution was prepared by diluting 2.72 mL 25% NH_4OH to 200 mL with phosphate buffer. The pH was measured with a pH meter (pH= 10.78 – 10.88, 21.5 ºC).

Host solution: A 0.1 mM host solution was prepared by dissolving 1.4 mg PA[6] (**4**) in 20 mL MeOH.

Guest solution: A 0.1 mM guest solution was prepared by dissolving 1.8 mg for guest 1, 1.2 mg for guest 2 or 1.2 mg for guest 3 in 20 mL of MeOH.

Immobilisation of the host: The prepared mITO electrodes were immersed in the host solution for 1.5 h. Afterwards, it was dipped for 5 min into MeOH and dried under an N_2 stream.

Guest binding: Three CV sweeps of the host-functionalised electrode are first measured (Figure S1), and afterwards, the host-modified electrode was soaked in a 0.1 mM guest solution for 16 to 18 h, followed by 5 min MeOH wash (10 mL).

Electrochemical methods

A CV (Figure S1) of the host-modified electrode (1.5 h soaking time) was measured in 0.2 M $NaClO_4$ (3 cycles, 0.2 to 1.2 V *vs.* NHE) before guest absorption to remove multilayers, which are formed during the host immobilisation on the surface.

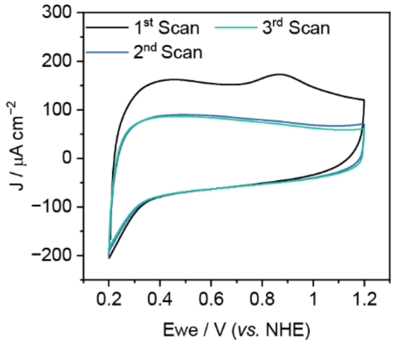

Figure S1. Electrode preparation, host-functionalised electrode in aqueous 0.2 M $NaClO_4$ (pH 7) at 100 mV s^{-1}.

Determination of the surface loading of the guest catalysts

The redox peak of the Ru^{II}/Ru^{III} oxidation is integrated with *EC-Lab* (*BioLogic* software) to give the loading in coulombs (C), which can be divided by the Faraday constant to give the moles of electrons. In the case of a broad redox peak (at pH 7 in 0.2 M $NaClO_4$ H_2O) this number is divided by two since the Ru^{III}/Ru^{IV} peak overlaps the Ru^{II}/Ru^{III} redox peak.

Surface loading of PA[6]

Desorption of the PA[6]

The UV/Vis absorption of five known concentrations of **4** in 1 M KOH in MeOH were measured to obtain a calibration curve at A = 304 nm with the equation y = 23378x + 0.0113 when the concentrations are given in mol L^{-1} or y = 0.023x + 0.011 in µM.

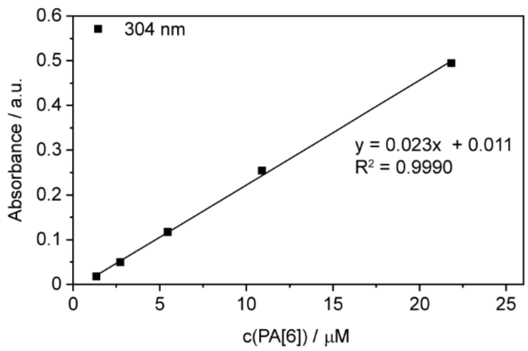

Figure S2. Calibration curve for PA[6] desorption in 1 M KOH in MeOH.

For the sample measurements, three mITO electrodes (3.3 to 3.6 cm^2) were functionalised with the host molecule **4** for 1.5 h, followed by MeOH wash and three CV sweeps, as described above. Afterwards, the plate is soaked for 1.5 h in 1 M methanolic KOH to desorb the Pillar[6]arene. The absorbance at 304 nm is measured and plugged into the aforementioned equation. Areas are geometric areas.

Table S1. PA[6] desorption from the surface with 1 M KOH MeOH.

sample	A (304 nm)	V/ mL	c (M)	Area /cm^2	Loading (mol/cm^2)
**p1**	0.02541	4.2	$1.57 \cdot 10^{-6}$	3.30	$1.999 \cdot 10^{-9}$
**p2**	0.01459	5.3	$1.10 \cdot 10^{-6}$	3.30	$1.779 \cdot 10^{-9}$
**p3**	0.03545	3.0	$2.00 \cdot 10^{-6}$	3.60	$1.667 \cdot 10^{-9}$

**Average**	1.81 ± 0.17 nmol cm^{-2}

Oxidative stability of the surface-bound pillar[6]arene

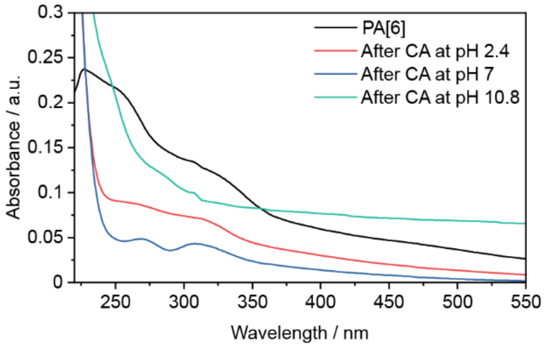

Figure S3. UV/Vis spectrum of the PA[6] in 1 M methanolic KOH before (black) and after desorption and applying catalytic conditions.

The surface-bound PA[6] was desorbed from the electrode with 1 M KOH in MeOH (by soaking it for 16 h) and the solution was measured with UV/Vis spectroscopy (Figure S3). A reference sample of the PA[6] is measured in solution (black) to compare the peaks with the desorbed species.

The host-modified PA[6] electrode was electrolysed for 30 min at 1 V *vs*. NHE in 0.2 M $NaClO_4$ (pH 7), and afterwards, PA[6] was desorbed from the electrode surface with 1 M methanolic KOH and measured with UV/Vis spectroscopy. The electrolysed sample at pH 7 (blue) and the initial one showed similar absorbance maxima, which indicated that the surface-bound PA[6] was not oxidised under these conditions.

We assume that the oxidation is hindered due to the binding to the surface. This also explains why, for multiple CV sweeps of the host-modified electrode, only the first cycle shows the oxidation peak of the PA[6], which can be attributed to the molecules forming multilayers on the surface and are not bound to the surface. The other cycles do not show any redox peak anymore, which should be present if the surface-bound macrocycle is oxidised (Figure S1).

To confirm this theory under catalytic conditions, we repeated the experiment with PA[6]-modified electrodes, operating them under the same conditions as our actual electrocatalysis. One sample was electrolysed at pH 10.8 (0.2 M NH_3 phosphate-buffered, CA for 90 min at 0.9 V *vs.* NHE) and one sample at pH 2.4 (0.1 Na_2SO_4, H_2SO_4, CA for 2 h at 1.7 V *vs.* NHE). Afterwards, the macrocycles were desorbed from the surface with 1 M KOH in MeOH and UV/Vis absorbance spectra were measured from the solutions (Figure S3, red and green). The sample which was electrolysed under acidic conditions showed a very similar absorbance spectrum to the PA[6] reference sample, which indicates that the PA[6] on the surface is not oxidised. For the sample that was oxidised under basic conditions (green), the peaks look similar but less strongly pronounced. This could be due to the loss of macrocycle into the anode solution during catalysis. We assume that, under both catalytic conditions the macrocycle is not oxidised on the surface. However, its desorption from the surface is favoured at high pH.

Theoretical calculations

Computational details

All computations were performed using the CP2K code.[57] The electronic structure was described using the Perdew-Burke-Ernzerhof (PBE) functional[58] with a non-local rVV10 dispersion interactions[59] within a Gaussian plane wave framework. The molecular orbitals were expanded using the TZV2P-MOLOPT-SR-GTH basis set[60] (In: 13, O: 6, C: 4, H: 1), while core electrons were described via the Goedecker-Teter-Hutter (GTH) pseudopotentials.[61] The basis sets were selected based on the partial density of states of the bulk structure of In_2O_3. The plane-wave basis set was truncated using a cut-off energy of 600 Ry. All calculations found convergence at an accuracy of 10^{-6} Hartree.

The surface of In_2O_3 (Ia-3) [206] was prepared from an optimised bulk structure (10.41 Å Literature: 10.18 Å[62]) using the Atomic Simulation Environment (ASE).[63] An 8 x 8 x 4 In atoms supercell of In_2O_3 (111) was constructed, and the bottom two layers were fixed upon optimisation. All surfaces were optimised using a vacuum layer of > 15 Å. For the optimisation of the adsorbate, the vacuum was applied above the height of the adsorbate.

Adsorption energies were calculated from the total energies of adsorbed molecules subtracted by the energies of the optimised molecules and substrate, where all three energy terms refer to fully optimised geometries.

$$E_{ads} = E_{\frac{surf}{mol}} - \left(E_{surf} - E_{mol}\right) \quad \text{(eq. 1)}$$

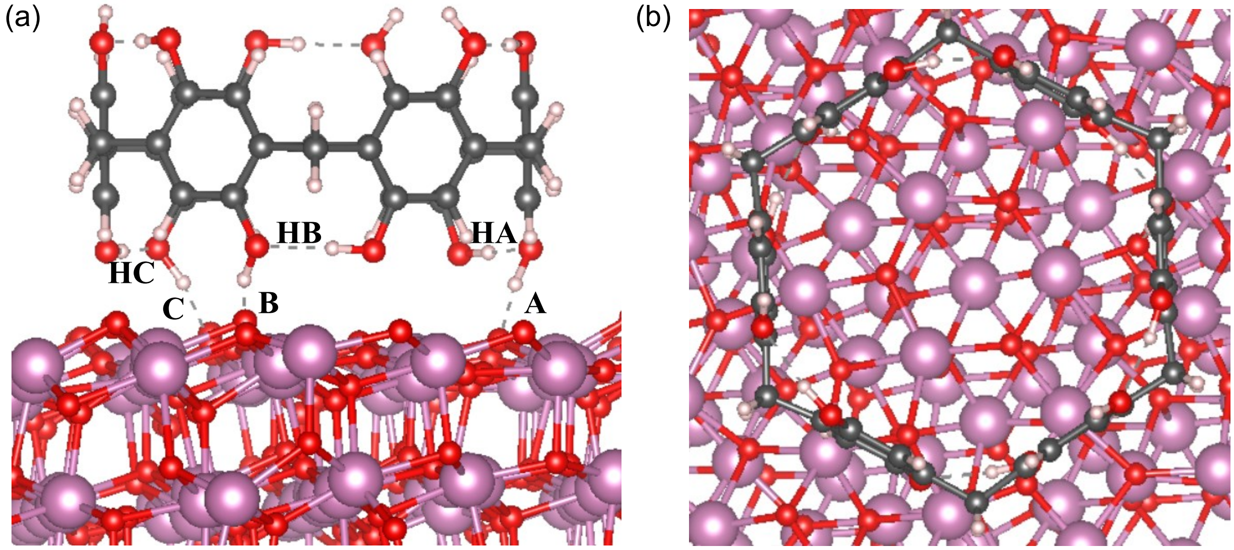

Figure S4. DFT optimised structures of **4**. Side view (a) and top view (b) of the optimised structure of the Pillar[6]arene on In_2O_3 (111). Atom colour code: In = purple; C = dark grey; O = red; H = white.

The interaction of the fully protonated Pillar[6]arene on In_2O_3 occurs via three hydrogen bonds (A, B, C) on the surface (Figure S4). Different bond lengths of the hydroxy units of the PA[6] to the oxygen atoms of the surface were observable. The resulting distances are A = 1.40 Å, B = 1.56 Å and C = 1.64 Å, corresponding to a total adsorption energy of -1.18 eV. Three intramolecular hydrogen bonds remain (HA, HB, and HC). We propose that the hydrogen-bonded state is a precursor of a reactive process that, via water elimination, leads to stronger chemical bonding, as suggested by the experimentally revealed high stability of the adsorbed molecule.

Figure S5 depicts the optimised structure of the adsorbed molecule after the elimination of two water molecules. The potential product was simulated by replacing two of the undercoordinated lattice oxygens with two oxygens of the PA[6]. After structure optimisation, the interaction energy amounts to -6.92 eV, indicating a strong binding to the surface.

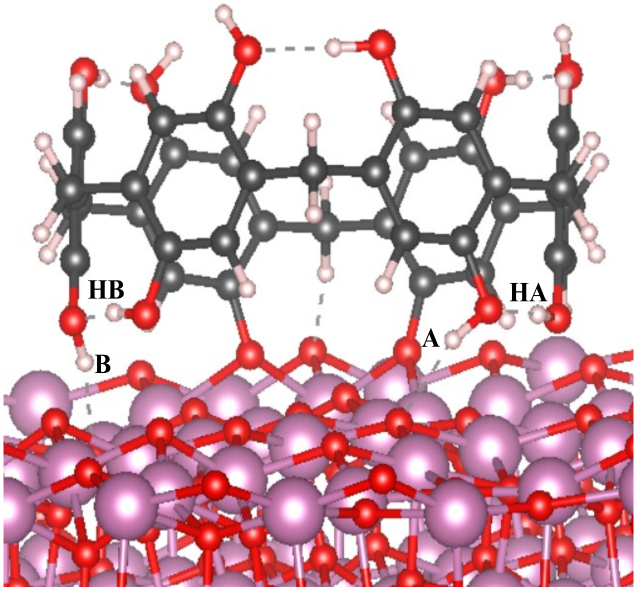

Figure S5. DFT optimised structures of **4**. Side view of the optimised structure of the Pillar[6]arene on In_2O_3 (111) after the elimination of two water molecules. Atom colour code: In = purple; C = dark grey; O = red; H = white.

Surface loading of the guest catalysts

Table S2. Calculation of the catalyst loading of Guest **1**, **2** and **3** at pH 2.4.

Catalyst	CV-Integral (mC)	Catalyst Loading (nmol)	Catalyst Loading (nmol cm^{-2})
Guest 1	0.610	6.32	2.11
Guest 1	0.624	6.47	2.16
Guest 1	0.571	5.92	1.98
Average	0.602 ± 0.023	6.24 ± 0.23	2.08 ± 0.08
Guest 2	0.618	6.40	2.13
Guest 2	0.551	5.72	1.91
Guest 2	0.563	5.83	1.94
Average	0.577 ± 0.028	5.98 ± 0.30	1.99 ± 0.10
Guest 3	0.636	6.59	2.20
Guest 3	0.583	6.04	2.02
Guest 3	0.599	6.21	2.07
Average	0.606 ± 0.022	6.28 ± 0.23	2.09 ± 0.08

Physisorption

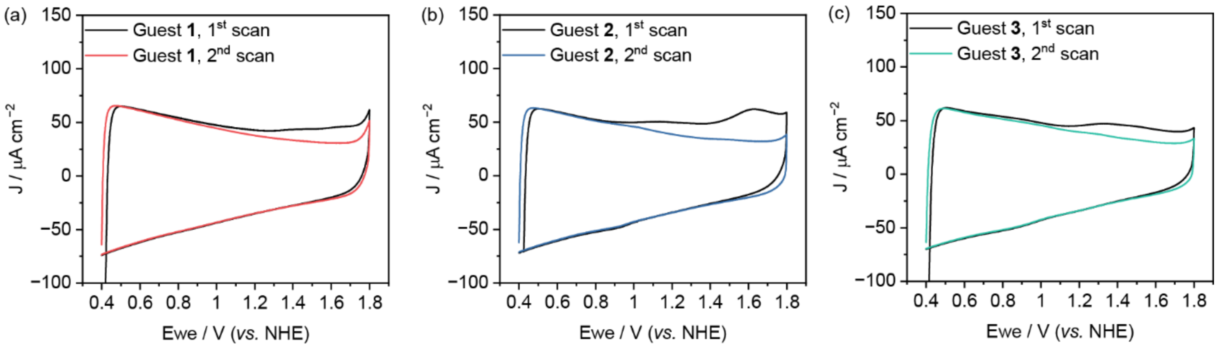

Figure S6. CV of the bare mITO electrode with physisorbed guest for 16 h in aqueous 0.1 M Na_2SO_4 (pH= 2.4) at 100 mV s^{-1}. (a) guest **1**, (b) guest **2** and (c) guest **3**.

The three guest molecules were separately physisorbed on an mITO electrode for 16 h each without previous host functionalisation. The physisorbed guest molecules are not bound to the surface and are removed within two CV scans (the first scan black and the second one in colour), demonstrating the host necessity of the host macrocycle for the guest binding to the surface.

Desorption of the guest

A host functionalised electrode (1.5 h in 0.1 mM **4** in MeOH) was absorbed with guest **1** for 1 h before a CV was measured in aqueous 0.2 M $NaClO_4$ before (solid line) and after (dashed line) desorption with DMSO. By soaking the host-guest electrode in DMSO for 90 min, the guest can be partially removed. This method can be used to exchange guest molecules without running electrocatalysis. The host remains bound to the surface only the guest molecule is removed with the DMSO washing.

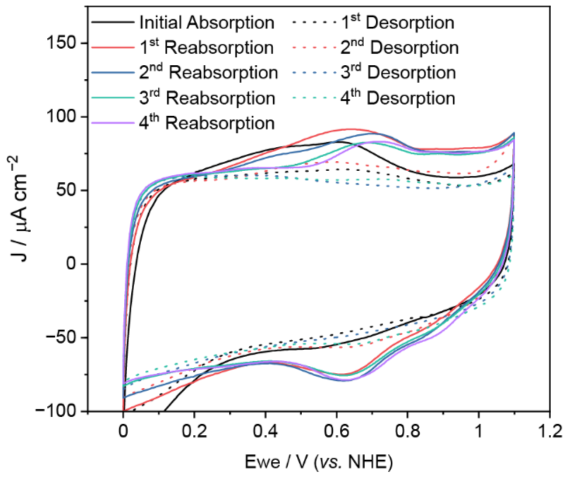

Figure S7. CV with host-guest modified electrode in aqueous 0.2 M $NaClO_4$, after absorption (solid line) and desorption (dashed line) of guest **1.**

Scan rate dependence

A working electrode was prepared according to the aforementioned procedure with PA[6] host, followed by 16 h of guest **1**. Afterwards, the scan rate dependence was measured using an aqueous 0.2 M $NaClO_4$ solution (pH 7). Plotting the peak current *vs.* the scan rate from 100 to 10 mV s^{-1} (100, 90, 80, 70, 60, 50, 20, 10 mV s^{-1}) shows a linear dependence, which is typical for surface-bound redox species. [40]

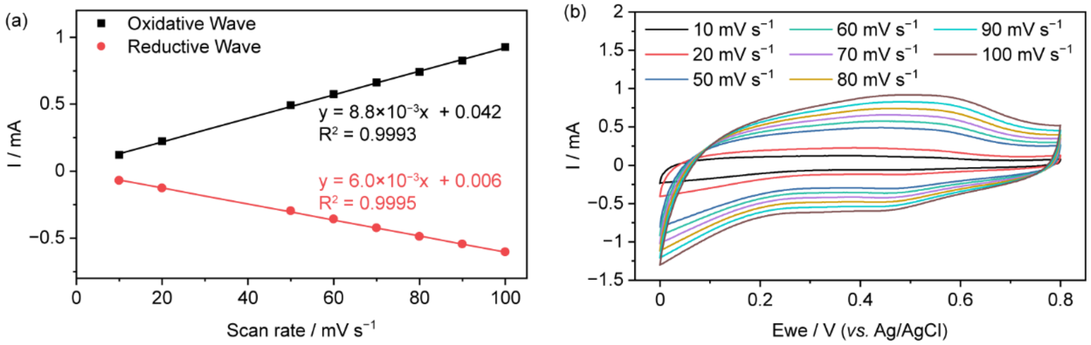

Figure S8. Scan rate dependence experiment (a) Current *vs.* scan rate plot (b) CVs in aqueous 0.2 M $NaClO_4$.

NMR Host-Guest binding studies

1.5 mg of PA[6] (**4**) was dissolved in 2 mL MeOD (1 mM), and 600 µL were used to measure the initial host NMR. The remaining 1.4 mL was used to dissolve the guest molecule.

For guest 1, 13.5 mg (0.0152 mmol); for guest 2, 9.80 mg (0.0157 mmol); and for guest 3, 9.5 mg (0.0152 mmol) were each dissolved separately in 1.4 mL host solution in MeOD (1 mM) and added stepwise to the host solution. A ^{1}H NMR spectrum is captured after every titration step, and the chemical shift change of the two host peaks at 6.46 ppm (aromatic CH) and 3.65 ppm (bridging CH_2) are monitored. The shift change was plotted with supramolecular *BindFit* to obtain the binding constants.[57]

An extreme peak broadening of the CH_2 peak is observable upon guest titration (especially for guest **3**), indicating a decreased molecular movement of the host molecule, which is assumed to be a consequence of the guest binding causing restricted molecular movement.

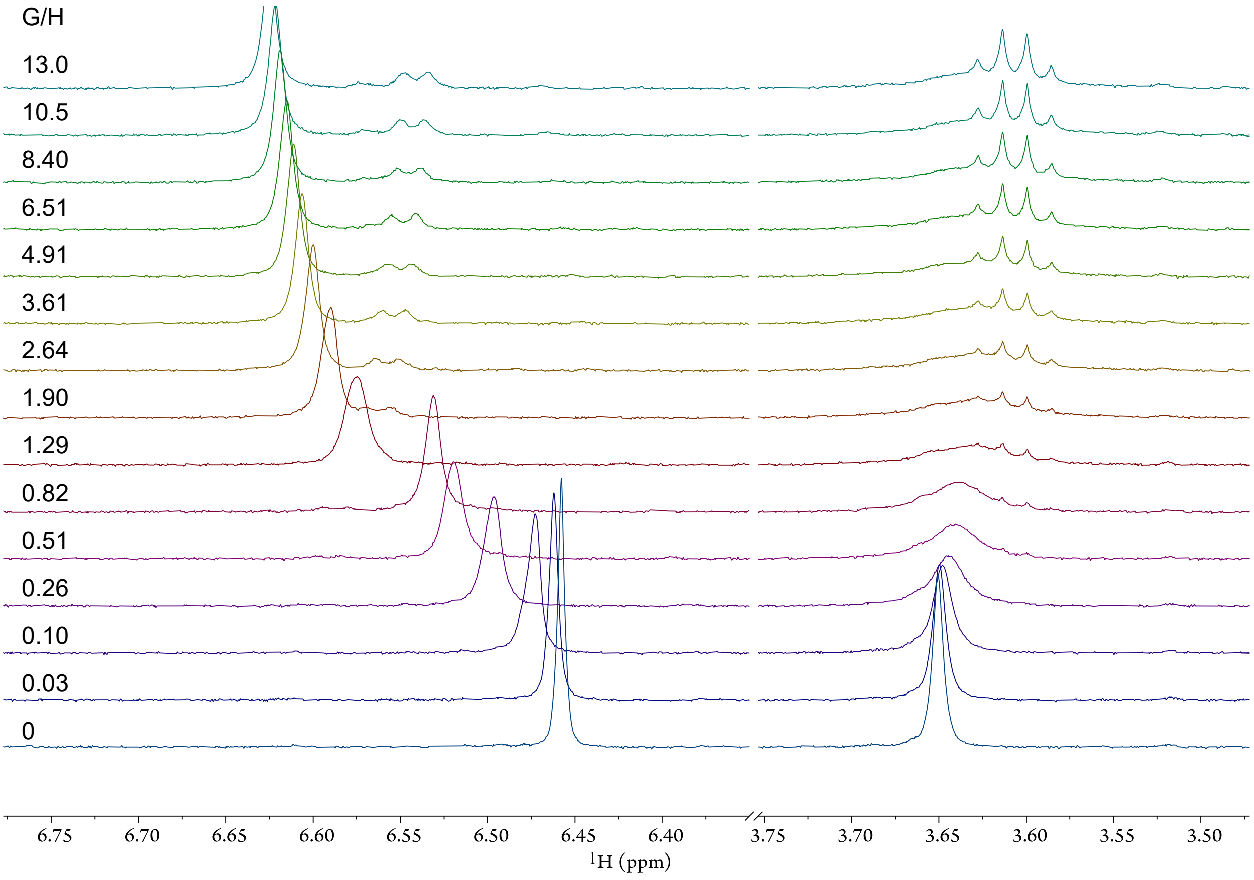

Figure S9. ^{1}H NMR spectra of the host-guest titration of PA[6] with guest **1**, increasing the guest concentration according to Table S3 from the bottom to the top, resulting in a binding constant of $K_{11} = 2891 \pm 391\ M^{-1}$.

Table S3. Data of the host-guest titration of PA[6] with guest **1**.

Host c(M)	Guest 1 c(M)	G/H equivalent total	y1: Shift, (ppm)	y2: Shift (ppm)
0,001024	0	0	6,45789	3,65068
0,001024	0,00003	0,03	6,46213	3,64941
0,001024	0,00010	0,10	6,47279	3,64798
0,001024	0,00027	0,26	6,49641	3,64400
0,001024	0,00052	0,51	6,51939	3,64003
0,001024	0,00084	0,82	6,53120	3,63812
0,001024	0,00132	1,29	6,57497	3,63272
0,001024	0,00194	1,90	6,58999	3,62810
0,001024	0,00270	2,64	6,60013	3,62747
0,001024	0,00370	3,61	6,60604	3,62286
0,001024	0,00503	4,91	6,61079	3,62079
0,001024	0,00667	6,51	6,61515	3,61761
0,001024	0,00860	8,40	6,61888	3,61427
0,001024	0,01078	10,5	6,62157	3,61364
0,001024	0,01326	13,0	6,62478	3,61221

Link to *BindFit* (guest 1):

http://app.supramolecular.org/bindfit/view/829135a8-b580-4bbf-acb9-79be3046f344

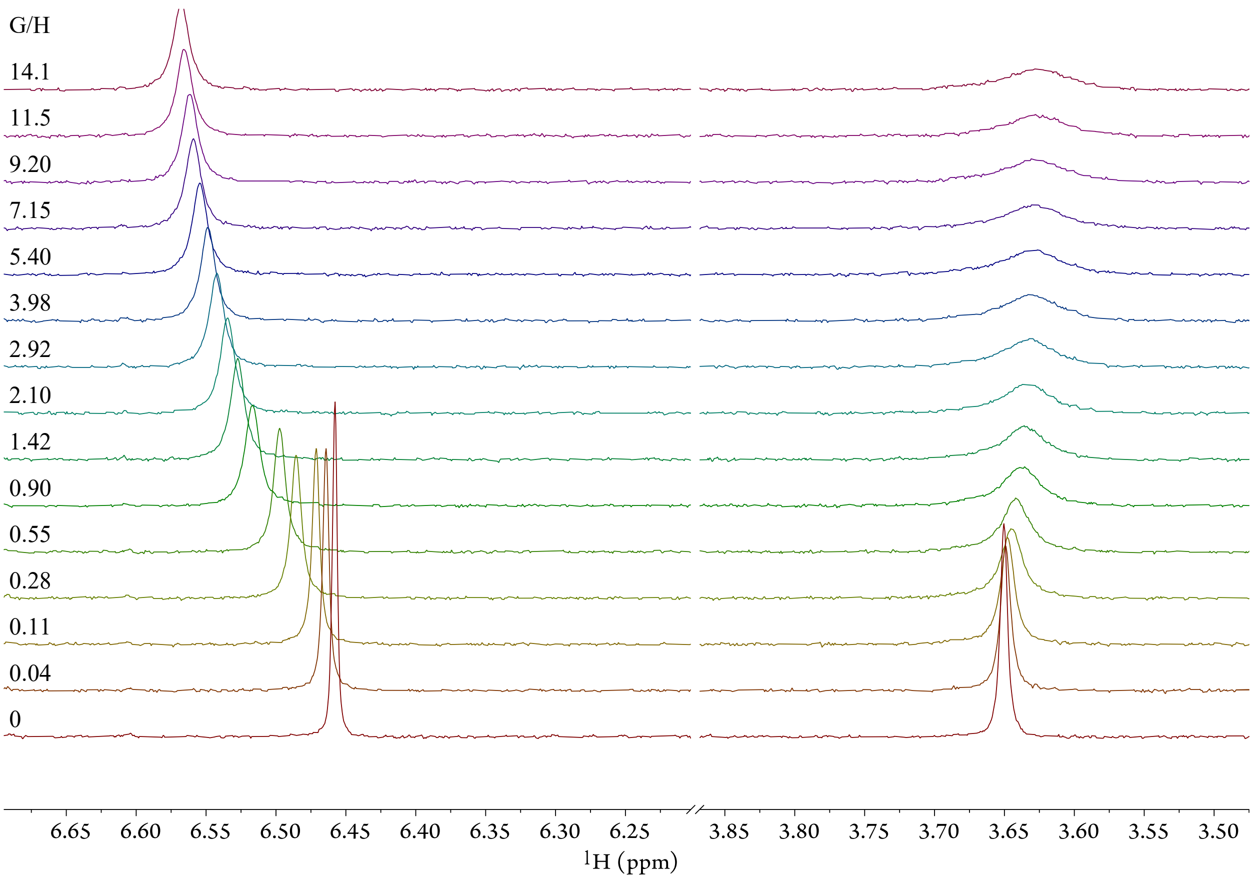

Figure S10. ^{1}H NMR spectra of the host-guest titration of PA[6] with guest **2**, increasing the guest concentration according to Table S4 from the bottom to the top, resulting in a binding constant of K_{11} = 2260 ± 362 M^{-1}.

Table S4. Data of the host-guest titration of PA[6] with guest **2**.

Host c(M)	Guest 2 c(M)	G/H equivalent total	y1: Shift, (ppm)	y2: Shift (ppm)
0,001024	0	0	6,457885	3,65032
0,001024	0,00004	0,04	6,464085	3,64913
0,001024	0,00011	0,11	6,471214	3,64782
0,001024	0,00029	0,28	6,485627	3,64480
0,001024	0,00056	0,55	6,497251	3,64156
0,001024	0,00092	0,90	6,516831	3,63758
0,001024	0,00145	1,42	6,527369	3,63334
0,001024	0,00215	2,10	6,534705	3,63035
0,001024	0,00299	2,92	6,542248	3,63035
0,001024	0,00408	3,98	6,548705	3,62911
0,001024	0,00553	5,40	6,554285	3,62811
0,001024	0,00732	7,15	6,559038	3,62712
0,001024	0,00942	9,20	6,561621	3,62612
0,001024	0,01178	11,5	6,565702	3,62512
0,001024	0,01445	14,1	6,567665	3,62413

Link to *BindFit* (guest 2):

http://app.supramolecular.org/bindfit/view/8496c545-6e17-4b86-9ac3-e99d0f05dc48

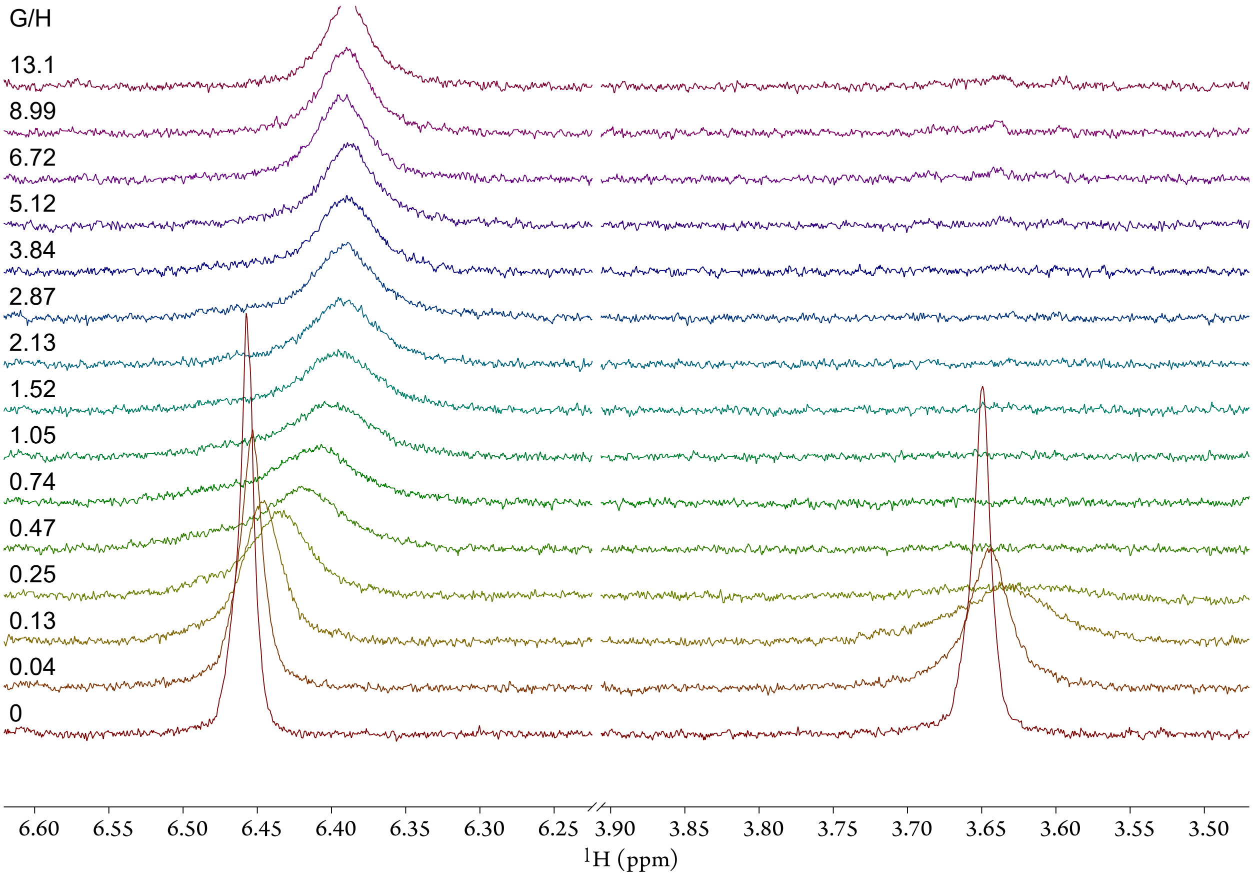

Figure S11. ^{1}H NMR spectra of the host-guest titration of PA[6] with guest **3**, increasing the guest concentration according to Table S5 from the bottom to the top, resulting in a binding constant of $K_{11} = 16492 \pm 11860\ M^{-1}$.

Table S5. Data of the host-guest titration of PA[6] with guest **3**.

Host c(M)	Guest 3 c(M)	G/H equivalent total	y1: Shift, (ppm)	y2: Shift (ppm)
0,001024	0	0	6,45758	3,64973
0,001024	0,00004	0,04	6,45362	3,64483
0,001024	0,00013	0,13	6,44631	3,63417
0,001024	0,00026	0,25	6,43524	3,62668
0,001024	0,00048	0,47	6,41863	3,62196
0,001024	0,00076	0,74	6,40874	3,61835
0,001024	0,00108	1,05	6,40024	3,61594
0,001024	0,00156	1,52	6,39431	3,60991
0,001024	0,00218	2,13	6,39292	3,60629
0,001024	0,00294	2,87	6,39095	3,60388
0,001024	0,00393	3,84	6,38838	3,60207
0,001024	0,00524	5,12	6,38998	3,60027
0,001024	0,00688	6,72	6,38949	3,59786
0,001024	0,00921	8,99	6,38884	3,59605
0,001024	0,01344	13,1	6,38786	3,59424

Link to *BindFit* (guest 3):

http://app.supramolecular.org/bindfit/view/596e69e7-50c5-4de2-9b74-4649ba873f87

NMR temperature studies

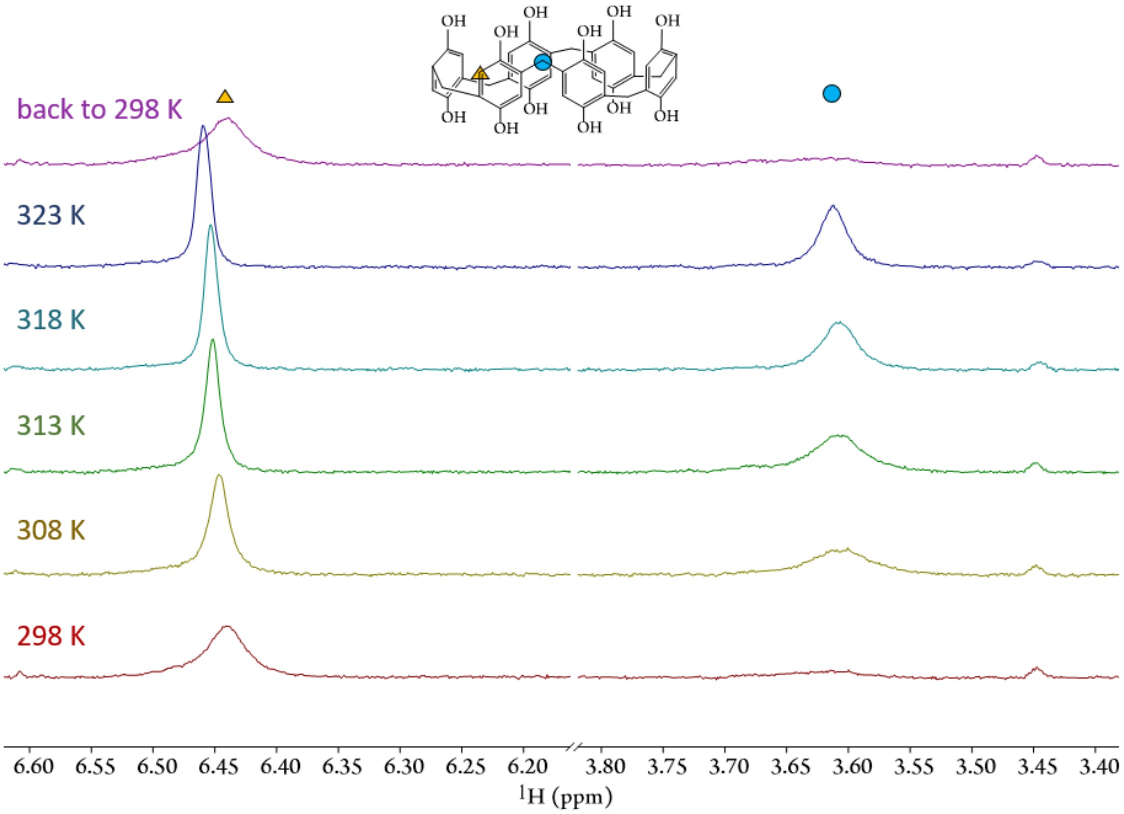

Figure S12. ^{1}H NMR temperature studies: 1 mM PA[6] (**4**) and 3.4 · 10^{-4} M guest **3** (ratio G:H = 0.23) at 298 K (red) and stepwise heating to 323 K (blue) and cooling back to 298K (violet), leading to a peak sharpening while increasing temperature and broadening when cooling back to RT (298 K). The effect is visible, especially for the CH_2 peak at 3.62 ppm.

The peak broadening indicates a very strong binding between the host and guest **3**, restricting the molecular movement of the host and leading to a short t_2 transverse relaxation time, which causes peak broadening. Upon heating the sample, the molecular movement increases, which enhances the transverse relaxation time t_2, followed by peak sharpening.

Ligand exchange

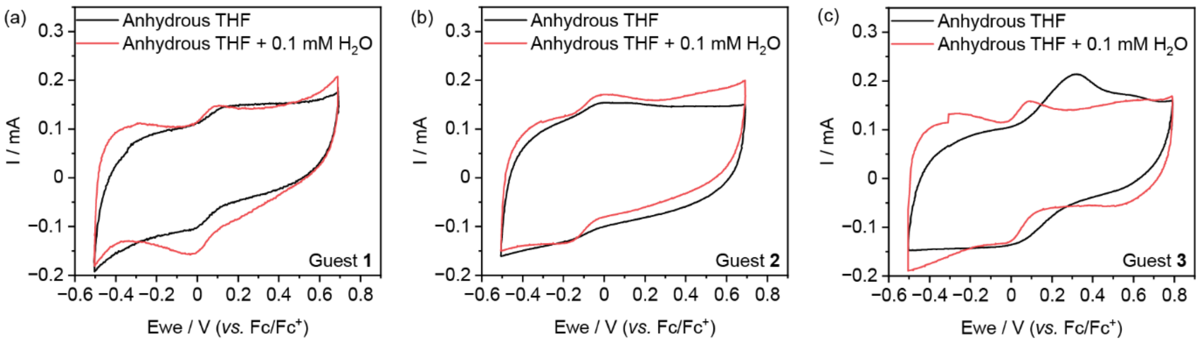

Figure S13. Ligand exchange experiment of 0.1 mM of (a) guest 1, (b) guest 2, and (c) guest 3 homogeneous in dry THF solution (0.1 M $TBAPF_6$), cyclic voltammetry was performed at 100 mV s^{-1} before (black) and after (red) the addition of water. Conditions: WE: mITO, RE: AgCl in 0.1 M $TBAPF_6$ THF, CE: Pt.

Ligand exchange experiments were performed homogeneously in 0.1 M $TBAPF_6$ in dry THF. For this, 0.1 mM of each guest molecule was dissolved in dry THF, and a CV at 100 mV s^{-1} was recorded under N_2 atmosphere (Figure S13). Afterwards, 300 µL H_2O were added to the mixture, and a CV was recorded again immediately. A peak shift to lower overpotentials is observable for all three complexes, which we assume is due to the ligand exchange of the chloride with an aqua ligand, causing a higher electron density at the Ru centre.

Pourbaix diagram of guest 1

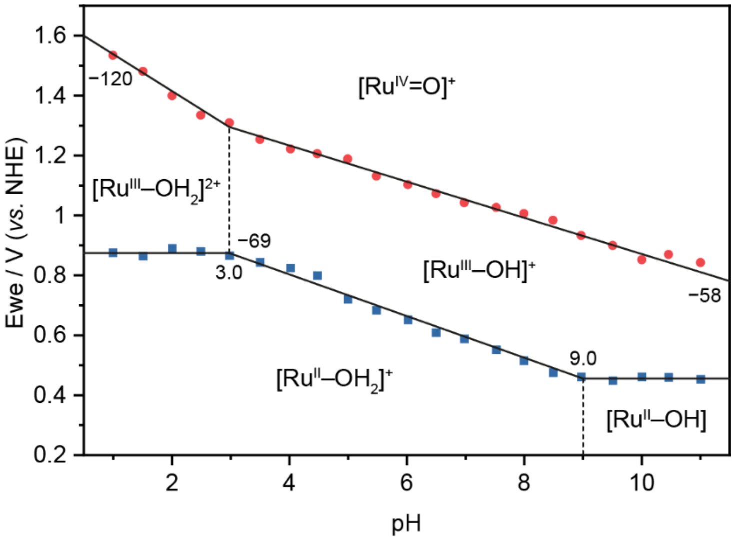

Figure S14. Pourbaix diagram of $[Ru(tpada)(bpy\text{-}NMe_2)(Cl)](PF_6)$ (**1**) immobilised on PA[6]-modified mITO in the pH range of 1.0–12.0 (pKa values are denoted by vertical dashed lines, slopes are given in mV pH^{-1}). Zones of stability for the different species are assigned with simplified structures and omission of additional ligands (e. g. $[Ru^{II}-OH_2]$ indicates $[Ru(tpada)(bpy\text{-}NMe_2)(H_2O)]^+$). Experimental conditions: Britton–Robinson buffer (0.1 M), pH was adjusted with an aqueous solution of NaOH (5 M), 100 mV s^{-1}.

pH Stability

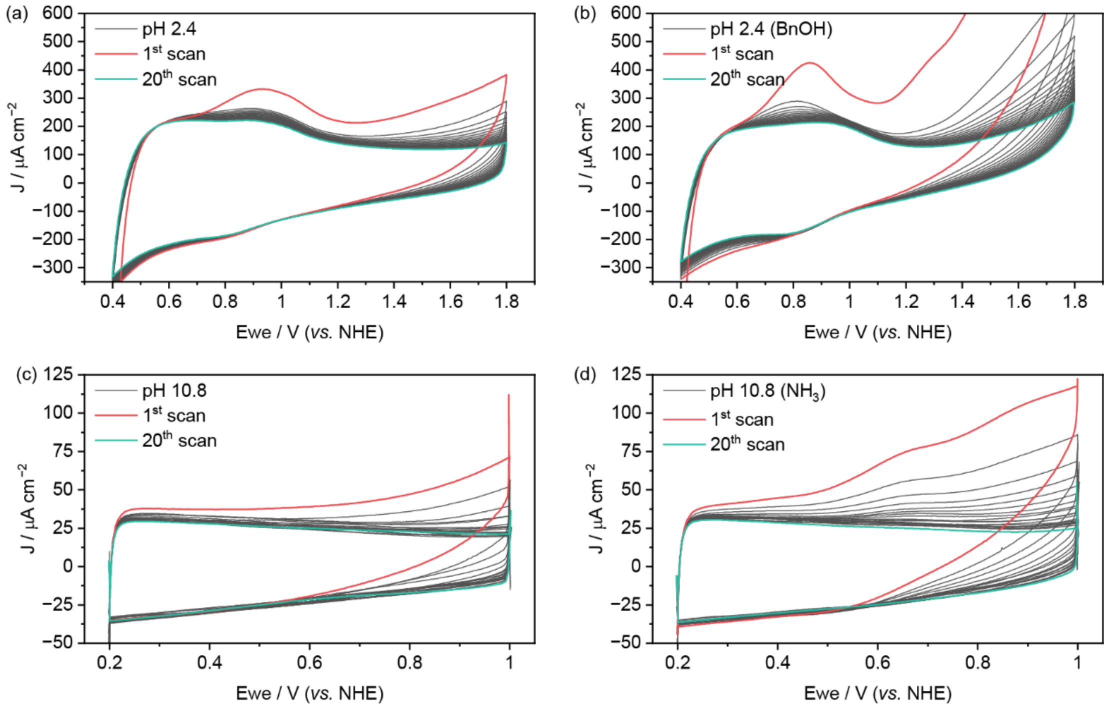

Figure S15. Stability test of the system under catalytic conditions: 20 CV sweeps with a mITO + host + guest 1 electrode were performed at different pH, with and without substrate: (a) 100 mV s^{-1} in 0.1 M Na_2SO_4 (drops of H_2SO_4 pH 2.4), (b) 100 mV s^{-1} in 0.1 M Na_2SO_4 (drops of H_2SO_4 pH 2.4) and benzyl alcohol (10 mM), (c) 20 mV s^{-1} in 0.1 M phosphate buffer (adjusted to pH 10.8 with KOH) and (d) 20 mV s^{-1} in 0.2 M NH_3 phosphate buffer (pH 10.8). The first scan is always shown in red, and the last one is in green. Conditions: WE: mITO + host + guest 1, RE: AgCl (3 M KCl), CE: Pt.

Alcohol oxidation

(a) Product spectrum after 2 h electrocatalysis, measured in $CDCl_3$ spiked with 20 μM DMSO-d_6 for quantification.

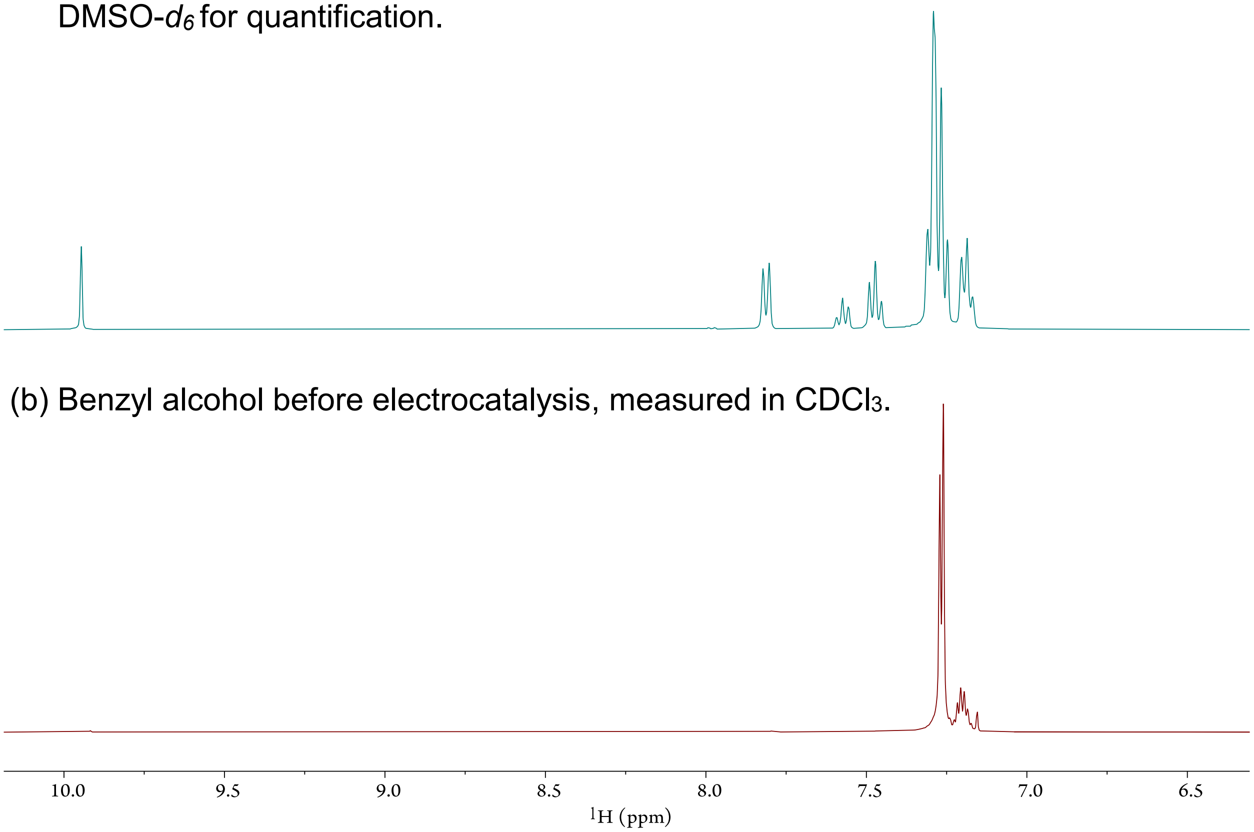

Figure S16. Example for an NMR spectrum of the (a) product after electrocatalysis and (b) starting material before electrocatalysis, zoomed into the aromatic region.

Quantification of products after alcohol oxidation

Reaction mixtures of chronoamperometry experiments with benzyl alcohol were extracted with $CDCl_3$ (1 mL), spiked with DMSO (7.10 uL) and dried over anhydrous $MgSO_4$. The qualitative and quantitative conversion of the starting materials was determined by ^1H NMR.

Table S6. Results table of control experiments of oxidation reactions with benzyl alcohol at pH 2.4.

Electrode Setup	Substrate	pH	Q (mC)
Guest 1 (Physisorption)	10 mM BnOH	2.4	15.7
Guest 2 (Physisorption)	10 mM BnOH	2.4	16.2
Guest 3 (Physisorption)	10 mM BnOH	2.4	29.0
Host + Guest 1	No Substrate	2.4	50.5
Host + Guest 2	No Substrate	2.4	46.5
Host + Guest 3	No Substrate	2.4	48.3
Host	10 mM BnOH	2.4	7.7
mITO	10 mM BnOH	2.4	21.5

Table S7. Full results table of oxidation reactions with benzyl alcohol at pH 2.4.

Catalyst	Absorption	pH	Q (mC)	FE (%)	NMR Yield (mM)	TON
Guest 1	1st	2.4	488	95.1	0.30	386
Guest 1	1st	2.4	630	90.4	0.37	473
Guest 1	1st	2.4	584	90.1	0.34	437
Guest 1	2nd	2.4	576	87.4	0.33	418
Guest 1	2nd	2.4	640	90.0	0.37	479
Guest 1	2nd	2.4	631	86.1	0.35	452
Guest 2	1st	2.4	416	94.5	0.26	341
Guest 2	1st	2.4	488	83.3	0.26	352
Guest 2	1st	2.4	449	86.6	0.25	337
Guest 2	2nd	2.4	454	94.5	0.28	372
Guest 2	2nd	2.4	598	91.6	0.36	475
Guest 2	2nd	2.4	515	90.2	0.30	402
Guest 3	1st	2.4	461	88.3	0.26	336
Guest 3	1st	2.4	428	91.6	0.25	324
Guest 3	1st	2.4	511	90.7	0.30	382
Guest 3	2nd	2.4	492	92.0	0.29	373
Guest 3	2nd	2.4	462	90.9	0.27	346
Guest 3	2nd	2.4	503	93.0	0.30	386

Table S8. Full results table of oxidation reactions with benzyl alcohol at pH 1.0.

Catalyst	Absorption	pH	Q (mC)	FE (%)	NMR Yield (mM)	TON
Guest 1	1st	1.0	440 ± 9	87.7 ± 3.0	0.25 ± 0.01	321 ± 4
Guest 1	2nd	1.0	335 ± 20	84.9 ± 4.1	0.19 ± 0.01	236 ± 3
Guest 2	1st	1.0	357 ± 6	84.3 ± 5.2	0.20 ± 0.01	261 ± 14
Guest 2	2nd	1.0	276 ± 18	87.4 ± 3.3	0.16 ± 0.01	209 ± 9
Guest 3	1st	1.0	386 ± 28	85.1 ± 2.5	0.21 ± 0.01	271 ± 14
Guest 3	2nd	1.0	296 ± 15	89.1 ± 5.2	0.17 ± 0.01	217 ± 16

Ammonia oxidation
Quantification of nitrate

Solution C was prepared by dissolving 172 mg (1 mmol) of sulfanilamide and 130 mg (0.5 mmol) of *N*-(1-naphthyl)ethylenediamine dihydrochloride in 10 mL of 37% HCl and, afterwards, diluting the mixture to 200 mL with H_2O. Subsequently, 470 mg (3 mmol) of VCl_3 was added to the mixture.[15,46] Solution C is stored in the fridge and can be used for up to 6 weeks. However, better results are obtained when the same colour solution is used for the calibration and the experiments. A peak shift of the absorption maximum is observable between the different batches of colour solutions (λ_{max} = 540 to 555 nm).

A serial dilution of a KNO_3 standard (10 mM) was done for the calibration. Afterwards, 2 mL of each concentration and 2 mL of colour solution C gave the following concentrations of 25, 10, 7.5, 2.5 and 1.25 µM KNO_3. The mixture was put in a water bath at (65˚C), let cool to RT and measured with UV/Vis against water as the baseline. The colour solution C has light blue staining and is diluted to half its concentration with 0.2 M NH_3 phosphate buffer (pH 10.8) and measured to find the contribution of the colour solution C to the sample measurements. The values are most precise when the reference sample is prepared and measured every time the experimental solutions are tested or is set as the baseline. Thus, variations of the colour solution and peak shifts can be monitored more easily. A calibration curve at λ_{max} = 540 nm with the correction from the colour solution blank gives the equation y = 23.682 + 0.011 with the concentration in mM, which was used to calculate the product concentrations obtained after electrocatalysis.

$$\mathbf{NH_4^+ + 3\ H_2O \longrightarrow NO_3^- + 10\ H^+ + 8\ e^-} \qquad \text{(eq. 2)}$$

With guest **1**, a selective conversion to nitrate was achieved, and no nitrite was detected.

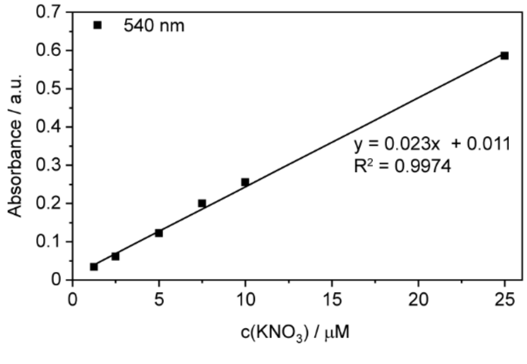

Figure S17. Calibration curve for the nitrate detection, at λ_{max} = 540 nm.

$$Absorbance\ sample = Absorbance - C_{blank} \qquad \text{(eq. 3)}$$

$$c_1 = \frac{Absorbance\ Sample - 0.011}{23.268} \quad \text{(eq. 4)}$$

$$c_2\ (NO_3^-) = \frac{c(mM)_1 * V_1}{V_2} = \frac{\mathbf{0.009}\left(\frac{mmol}{L}\right) \times 0.00402\ L}{0.002\ L} = 0.0017\ mmol/L \quad \text{(eq. 5)}$$

c_1 is the concentration of NO_3^- after dilution with the colour reagent (2 mL) and HCl (20 µL) solutions, which is then used to calculate c_2, the concentration of nitrate in the anode solution.

V_1= V_2 (2 mL anode solution) + color reagent C (2 mL)+ HCl (20 µL)

$$n\ (NO_3^-) = c_2 \times V_{anode\ solution} = 0.0017\left(\frac{mmol}{L}\right) \times 0.0072\ L = 1.24 \cdot 10^{-5}\ mmol \quad \text{(eq. 6)}$$

$$\boldsymbol{Q = nFN} \quad \text{(eq. 7)}$$

Q = charge passed (C); n = number of electrons (8); N = mol

$$\boldsymbol{Q}_{NO_3^-} = 8 \times 96485.33\left(\frac{C}{mol}\right) \times 1.24 \cdot 10^{-8}\ mol = 0.0096\ C$$

$$\textbf{FE} = \frac{Q_{NO_3^-}}{Qelectrochemical} \times 100 = \frac{(0.0096\ C)}{0.0098\ C} \times 100 = 98\% \quad \text{(eq. 8)}$$

Table S9. Results table for three guest **1** samples after 90 min CA in 0.2 M NH_3 (phosphate buffered, pH 10.8) at 0.9 V *vs.* NHE.

Guest 1	1	2	3	average
**$V_{Anode\ solution}$/ V_2 / V_1 (mL)**	7.2/ 2/ 4.02	7.5/ 2/ 4.02	7.4/ 2/ 4.02	
**$A_{Absorbance}$ (after correction)**	0.031	0,040	0,041	
**$c_2(NO_3^-)$ (mmol/L)**	0.0017	0,0025	0,0026	
**$Q_{electrochemical}$ (C)**	0.010	0.015	0,014	
**FE (%)**	98	98	104	**100** ± **4%**
**Surface loading (mol)**	$5.5 \cdot 10^{-9}$	$6.2 \cdot 10^{-9}$	$6.14 \cdot 10^{-9}$	
**Surface area (cm²)**	3.7	3.7	3.7	

Table S10. Reference measurements for ammonia oxidation for 90 min CA in 0.2 M NH_3 (phosphate buffered, pH 10.8) at 0.9 V *vs.* NHE.

	physisorbed	only mITO	only host	without substrate*
**$A_{Absorbance}$** (after correction)	BDL	BDL	BDL	---
**Surface area / cm²**	3.3	3.8	3.3	3.4
**$Q_{electrochemical}$ (C)**	$2.6 \cdot 10^{-3}$	$1.3 \cdot 10^{-3}$	$3.7 \cdot 10^{-3}$	$4.19 \cdot 10^{-3}$

BDL = below detection limit

*CA for 90 min at 0.9 V *vs.* NHE was performed with an mITO + host + guest 1 electrode in phosphate buffered (0.1 M) solution adjusted to pH 10.8 with KOH, without the substrate NH_3

Currents below 0.01 C lead to an undetectably small amount of nitrate. The quantification is limited by the detection limit of the Griess test and its relatively large error, which is also influenced by the absorption maximum peak shift of the colour solution at very low concentrations.

Reabsorption studies

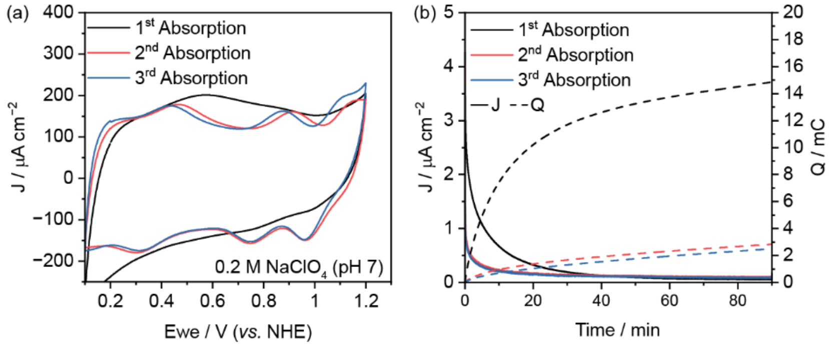

Figure S18. Reabsorption studies with guest **1** after 90 min electrocatalysis in 0.2 M NH_3 phosphate buffered solution at pH 10.8 (a) CV in 0.2 M aqueous $NaClO_4$ (pH 7, 100 mV s^{-1}) and (b) CA in 0.2 M NH_3 phosphate buffered solution (pH 10.8).

The first absorption shows a broad average peak, attributed to the overlapping of the Ru^{II}/Ru^{III} and the Ru^{III}/Ru^{IV} oxidations. After 90 min of electrocatalysis in 0.2 M NH_3 phosphate buffered solution at pH 10.8, reabsorption of the guest molecule is still possible, even though the surface loading is reduced to only 30% of its initial coverage, resulting in lower currents during the second electrocatalysis (red). We assume that PA[6] is lost during the first catalysis cycles, showing lower catalyst loading. Afterwards, the host on the surface remains stable since the third absorption (blue) is very similar to the second one (red). At this low current, product formation was below the detection limit of the Griess test.

Ammonia oxidation at higher pH

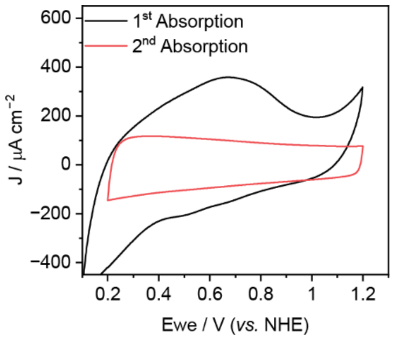

Figure S19. CV (100 mV s^{-1}**)** of guest **1** before (black) electrocatalysis at 0.97 V *vs.* NHE in 0.2 M NH_3 solution (pH 11.3) and reabsorption with fresh guest for 16 h (red) indicated that no reabsorption is possible after electrocatalysis at pH 11.3.

Stability of the mITO electrode at different pH

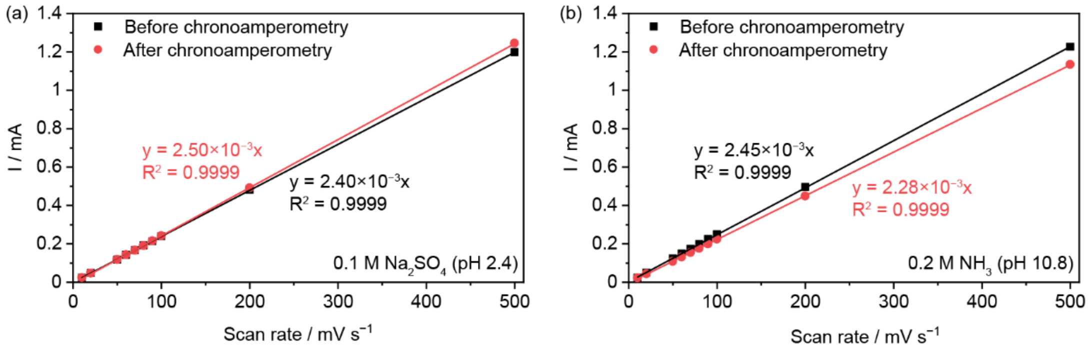

Figure S20. Surface area determination before and after chronoamperometry for (a) 1.5 h at 0.9 V *vs.* NHE at pH 10.8 (0.2 M NH_3 phosphate buffered) and (b) for 2 h at 1.5 V *vs.* NHE at pH 2.4 (0.1 M Na_2SO_4, acidified with 3 drops of H_2SO_4). CV sweeps at different potentials (500, 200, 100, 90, 80, 70, 60, 50, 20, 10 mV s^{-1}) of the mITO electrode are performed at pH 7 (0.2 M $NaClO_4$ in H_2O) and the current(the current at 0.5 V vs AgCl was used) is plotted vs the scan rate.

To test the stability of the bare mITO under the reaction conditions, the surface area was determined before and after chronoamperometry for 1.5 h at 0.9 V *vs.* NHE at pH 10.8 (0.2 M NH_3 phosphate buffered) and for 2 h at 1.5 V *vs.* NHE at pH 2.4 (0.1 M Na_2SO_4, acidified with drops of H_2SO_4). For that CV sweeps at different potentials (500, 200, 100, 90, 80, 70, 60, 50, 20, 10 mV s^{-1}) of the mITO electrode are performed at pH 7 (0.2 M $NaClO_4$ in H_2O) and the current is plotted *vs*. the scan rate (Figure S20). The same is done for FTO without mITO to determine the active surface area with mITO. By dividing the slope of the mITO by the slope of the bare FTO, a factor between 48 to 53 per cm^2 is calculated, giving a surface area for each sample before and after CA at different pH. The active surface area of the mITO electrode after CA at pH 10.8 showed that still 92% of the mITO are active in case of high pH, and the same area (104%) after CA at pH 2.4.

Table S11. Surface areas of the electrode before and after CA at low and high pH.

The surface area before CA at pH 10.8	178.8 cm^2
The surface area after CA at pH 10.8	164.5 cm^2
The surface area before CA at pH 2.4	171.6 cm^2
The surface area after CA at pH 2.4	178.8 cm^2

Guest, pH and medium exchange

PA[6]-modified electrodes can be reused for different catalytic reactions, including the exchange of guest molecules. To demonstrate that the PA[6]-modified electrodes are reusable for different reactions and at different pH after electrocatalysis and reabsorption with fresh guest, four electrodes (E1-E4) were prepared: Two electrodes (E1 and E2) were used to exchange the guest molecules to perform different reactions at different pH.

For the other two electrodes (E3 and E4), the same guest molecule was used to perform different reactions at different pH.

Ammonia oxidation is always performed for 90 min at 0.9 V *vs.* NHE in 0.2 M NH_3 phosphate buffered solution at pH 10.8. Alcohol oxidation is always performed for 2 h at 1.7 V *vs.* NHE in aqueous 0.1 M Na_2SO_4 at pH 2.4.

Guest **1** was used for alcohol oxidation and ammonia oxidation. Guest **3** was used for alcohol oxidation only.

Guest exchange to perform different reactions at different pH with the same electrode:

E1 and E2: Two host-modified electrodes were first absorbed with guest **3** (E1) or guest **1** (E2) for 16 h. Alcohol oxidation was performed with guest **3** (E1), followed by reabsorption of guest **1** to perform ammonia oxidation, and *vice versa* for E2, first performing ammonia oxidation with guest **1**, followed by reabsorption with guest **3** and alcohol oxidation.

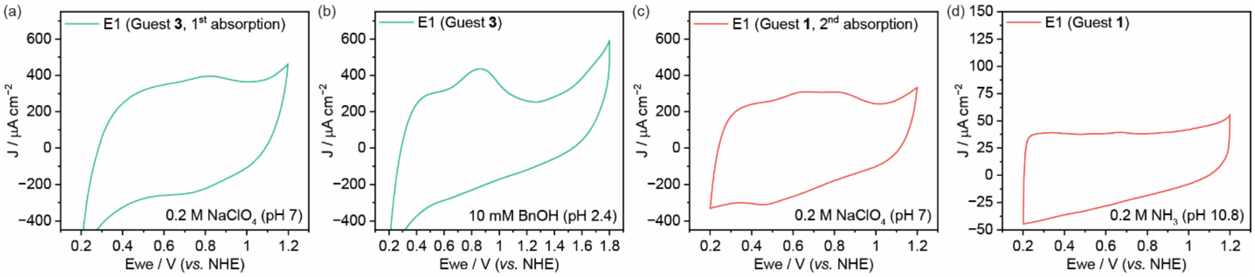

Figure S21. CV of the 1st absorption of (guest **3**) in (a) aqueous $NaClO_4$ (0.2 M, pH 7, 100 mV s^{-1}) and (b) CV upon addition of benzyl alcohol (10 mM). After electrocatalysis (alcohol oxidation with guest **3** on E1, entry E1, Table S13) guest **1** is reabsorbed onto the same electrode E1 (c) CV of 2nd absorption with guest 1 in aqueous $NaClO_4$ (0.2 M, pH 7, 100 mV s^{-1}) and (d) in aqueous NH_3 (0.2 M, phosphate buffer at pH 10.8, 20 mV s^{-1}) to then perform ammonia oxidation (entry E1, Table S12).

The same process can be performed the other way round, first performing ammonia oxidation with guest **1**, followed by reabsorption with guest **3** and alcohol oxidation as shown in Figure S22.

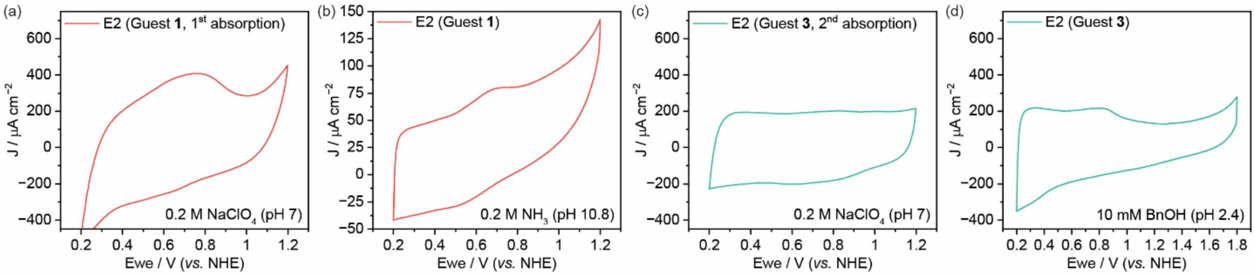

Figure S22. CV of the 1st absorption of (guest **1**) in (a) aqueous $NaClO_4$ (0.2 M, pH 7, 100 mV s^{-1}) and (b) in aqueous NH_3 (0.2 M, phosphate buffer at pH 10.8, 20 mV s^{-1}). After electrocatalysis (ammonia oxidation with guest **1** on E2, entry E2, Table S12) guest **3** is reabsorbed onto the same electrode E2. (c) CV of 2nd absorption with guest 3 in aqueous $NaClO_4$ (0.2 M, pH 7, 100 mV s^{-1}) and (d) CV upon addition of benzyl alcohol (10 mM), to then perform alcohol oxidation (entry E2, Table S13).

It was observed that the PA[6] is more stable at lower pH, which is why electrodes first used at lower pH (E1) work better for reabsorption (stronger redox peak) than if they were first used at high pH (E2). We assume that is due to the more stable binding of the host molecule at lower pH.

We showed that the guest can be exchanged, and the same electrode can be used to reabsorb a different guest molecule. Moreover, the same guest molecule can be reabsorbed to perform different reactions by only changing the substrate (pH).

Substrate (pH) exchange to perform different reactions with the same guest:

E3 and E4: Two host-modified electrodes were first absorbed with guest **1** each for 16 h. Afterwards, one electrode was used to perform ammonia oxidation (Table S12, entry E3), followed by reabsorption with the same guest **1** to perform alcohol oxidation (Table S12, entry E3), and vice versa (first Table S13, entry E4 then Table S12, entry E4).

Again, electrodes that were first used for alcohol oxidation perform better than electrodes that were first used for ammonia oxidation.

Table S12. Results for reabsorption and ammonia oxidation with guest **1** after 90 min CA at 0.9 V *vs.* NHE.

Electrode (G1 absorption)	E1 (2nd)	E2 (1st)	E3 (1st)	E4 (2nd)
$V_{Anode\ solution}$ / V_2 / V_1 (mL)	5.8/ 2/ 4.02	6/ 2/ 4.02	10/ 2/ 4.02	10/ 2/ 4.02
$A_{Absorbance}$ (after correction)	0.047	0.045	0.065	0.019
$c_2(NO_3^-)$ (mmol/L)	0.0032	0.0029	0.0047	0.0007
$Q_{electrochemical}$ (C)	0.0145	0.047	0.042	0.007
FE (%)	98	29	87	74
Surface loading (mol)	$6.1 \cdot 10^{-9}$	$9.89 \cdot 10^{-9}$	$8.16 \cdot 10^{-9}$	$5.7 \cdot 10^{-9}$
Surface area (cm^2)	3.7	3.8	3.8	3.8

Table S13. Results table of oxidation reactions with benzyl alcohol at pH 2.4.

Electrode	Catalyst	Absorption	pH	Q (mC)	FE (%)	NMR Yield (mM)	TON
**E1**	Guest 3	1st	2.4	458	91.3	0.27	362
**E2**	Guest 3	2nd	2.4	283	91.0	0.17	223
**E3**	Guest 1	2nd	2.4	337	89.9	0.19	259
**E4**	Guest 1	1st	2.4	550	92.9	0.33	443

Synthesis and Characterisation

Synthesis of [Ru(tpada) (pic)(Cl)] (**2**)

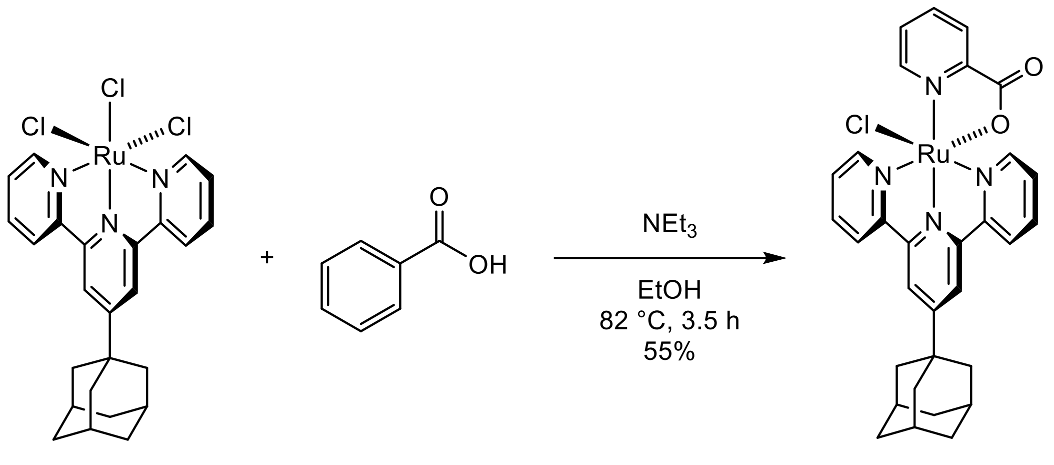

1b **2**

$RuCl_3$ 3 H_2O (451.53 mg, 2.18 mmol, 2 eq.) and terpyridine adamantane **1a**[15] (400 mg, 1.09 mmol, 1 eq.) were dissolved in 60 mL EtOH and stirred at reflux (82 °C) for 3 h. The solution was allowed to cool down to RT while a brown precipitate formed, which was filtered off to receive the product [Ru(tpada)$(Cl)_3$] **1b**[12] as a light-brown powder (612 mg, 1.06 mmol, 98%).

[Ru(tpada)$(Cl)_3$] (360 mg, 0.63 mmol, 1 eq.), picolinic acid (77.1 mg, 0.63 mmol, 1 eq.) and NEt_3 (131 µL µL, 0.94 mmol, 1.5 eq.) were dissolved in EtOH (50 mL) and stirred at reflux (82 °C) for 3.5 h. The residual solvent was evaporated, and the crude product was purified by column chromatography with an eluent system of CH_2Cl_2/MeOH (9:1, R_f = 0.68) to receive [Ru(tpada)(pic)(Cl)] (216 mg, 0.28 mmol, 55%) as a dark purple powder.

^{1}H NMR (500 MHz, DMSO-d_6, ppm): δ 9.88 (d, J = 4.5 Hz, 1H; pico-N-Ar-C**H**, 34), 8.80 (dt, J = 8.1, 1.3 Hz, 2H; pico-Ar-C**H**, 31,33), 8.64 (s, 2H; terpyridine-C**H**, 7,11), 8.24–8.03 (m, J = 7.7 Hz, 3H; pico-Ar-C**H**, 32 and terpyridine-Ar-C**H**: 6,18), 7.93 (td, J = 7.8, 1.5 Hz, 2H; (terpyridine-Ar-C**H**: 1,17)), 7.88 (dd, J = 5.6, 0.8 Hz, 2H; terpyridine-Ar-C**H**: 3,15), 7.52–7.42 (m, 2H; pico-Ar-H 2,16), 2.20 (s, 9H, **C**H and C**H₂**; adamantane-H: 19,21,23,25,27,28), 1.93–1.82 (m, 6H, C**H₂**; adamantane-H: 20,22,24).

^{13}C NMR (126 MHz, DMSO-d_6, ppm) δ 171.74 (C=O, 36), 159.82 (terpyridine-**C**: 12), 158.73 terpyridine-**C**: 8,10), 155.01 (terpyridine-**C**: 5,13), 152.32 (pico-Ar**C**-COO, 30),151.52 (terpyridine-Ar-**C**H: 3,15), 150.75 (pico-N-Ar-**C**H, 34), 135.36; 135.89; 127.91; 125.94 (pico-Ar-**C**H, 32 and terpyridine-Ar-**C**H: 6,18), 126.80 (m, 2H, pico-Ar-H 2,16), 122.78 (terpyridine-**C**H, 31,13), 118.36 (terpyridine-CH, 7,11) 41.85 (**C**H_2, 25, 27, 28), 36.00 (**C**H_2 and **C**, 20,22,24,26), 28.27 (**C**H bridging atoms, 19,21,23).

HRMS (ESI) m/z calcd for $C_{31}H_{29}ClN_4O_2Ru^+$ 626.1017 $[M]^+$; found 626.1022; calcd for $C_{31}H_{29}N_4O_2Ru^+$ 591.1329 $[M – Cl]^+$ found 591.1309

(a)

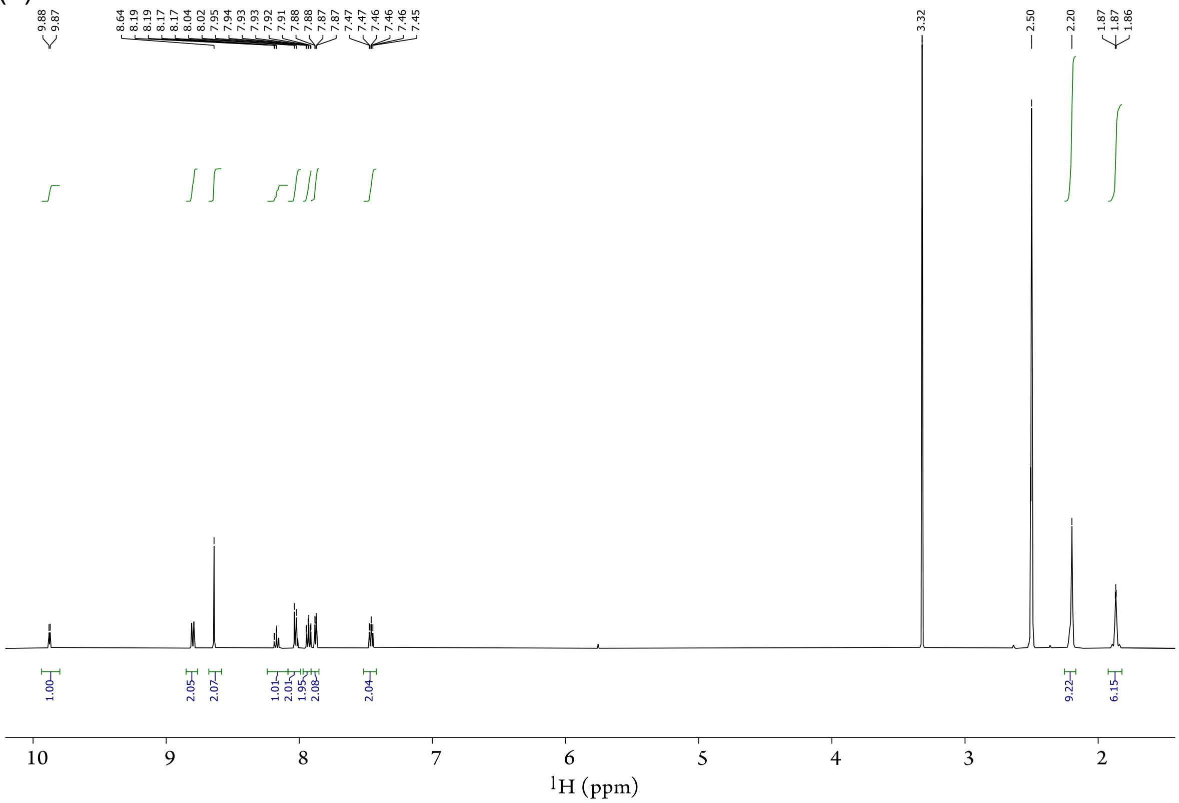

(b)

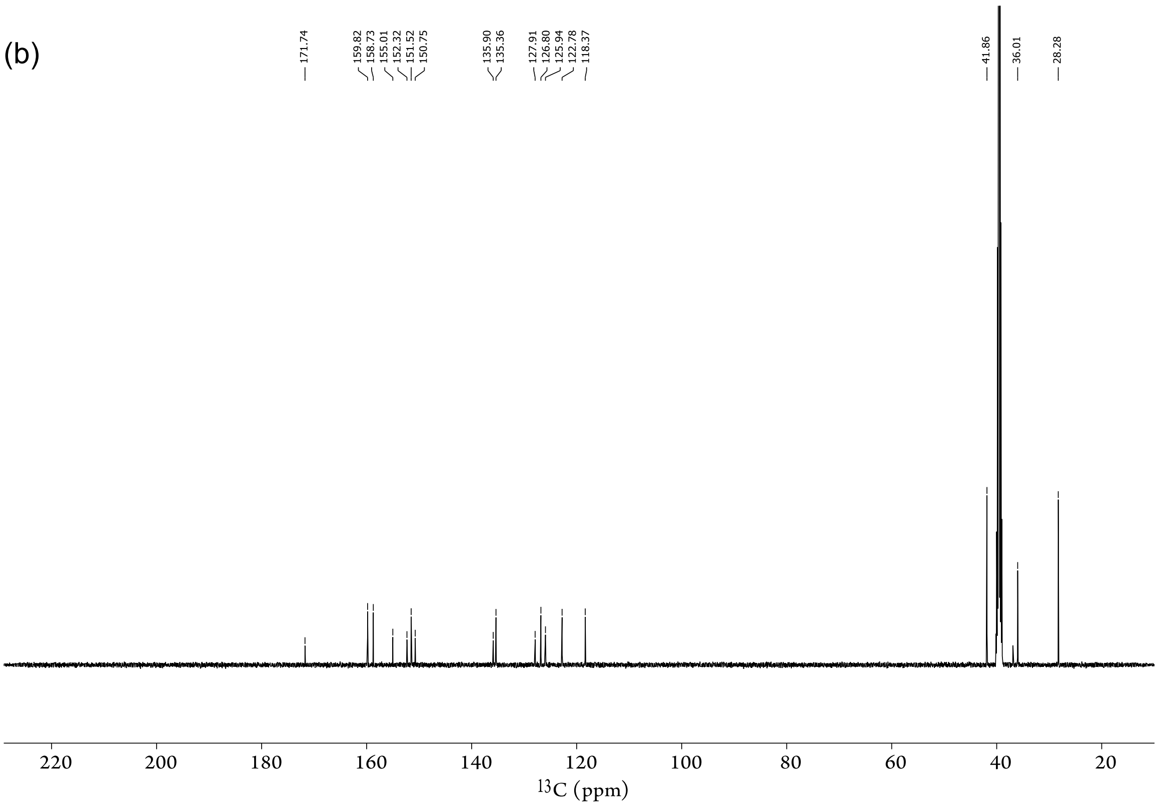

Figure S23. (a) ^{1}H and (b) ^{13}C NMR spectra of **2** in DMSO-d_6.

(a)

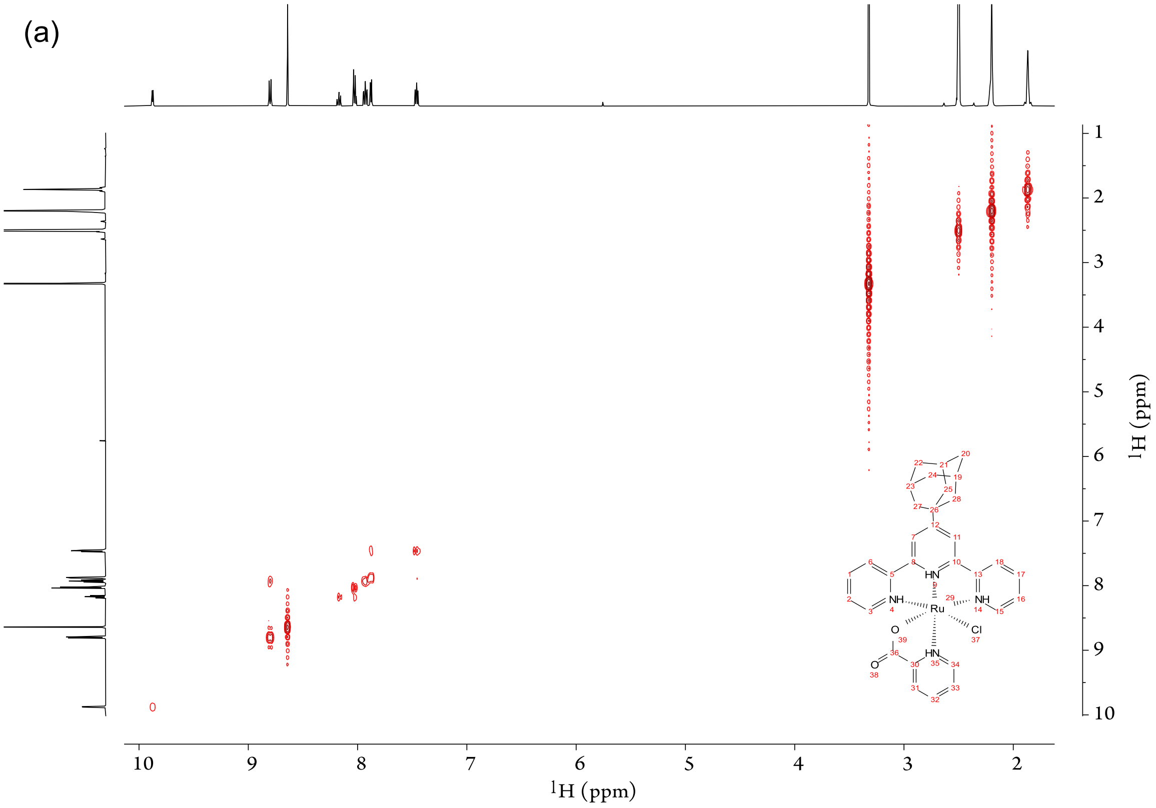

(b)

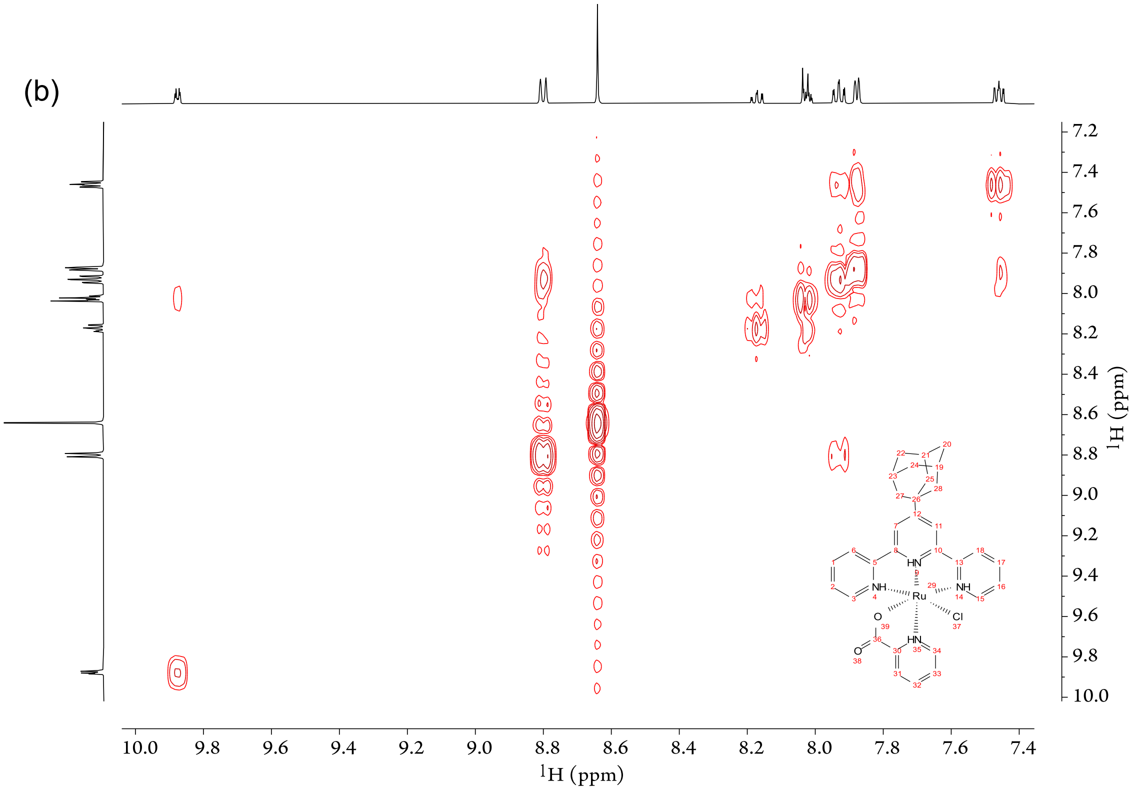

Figure S24. (a) Full spectrum (b) zoomed in aromatic region COSY (^1H – ^1H) NMR spectra of **2** in DMSO-d_6.

(a)

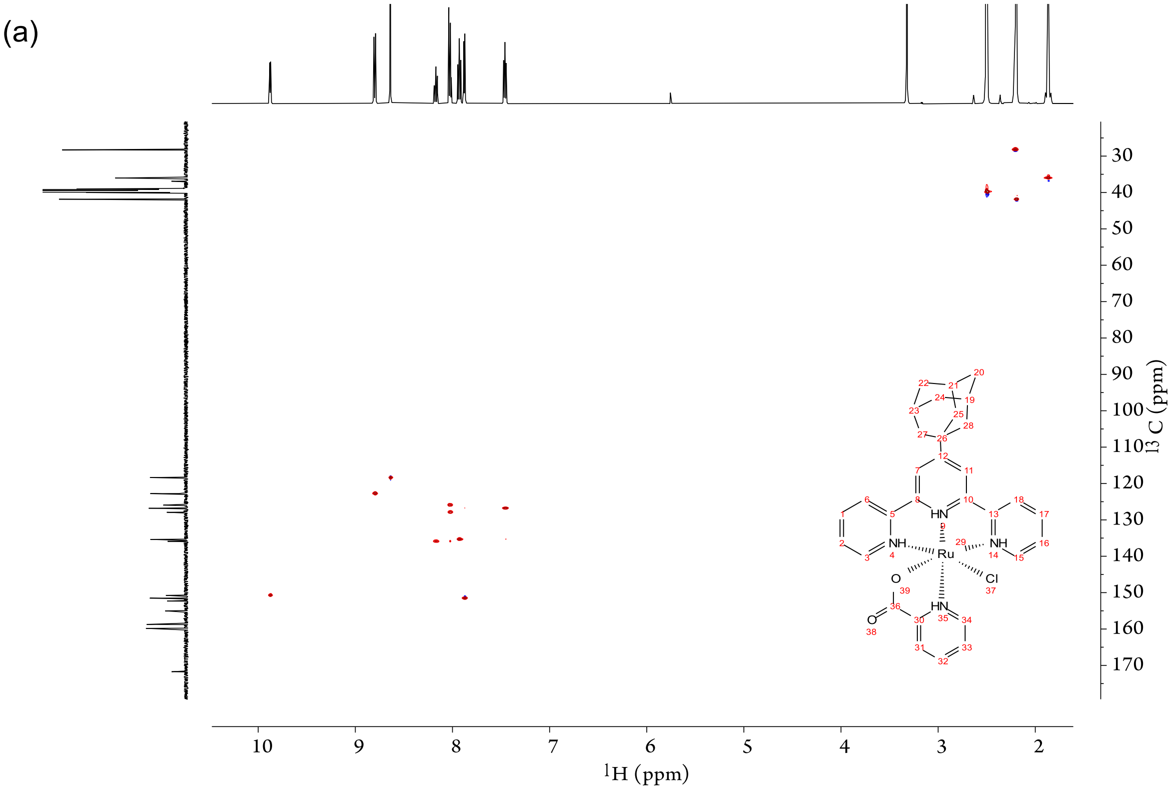

(b)

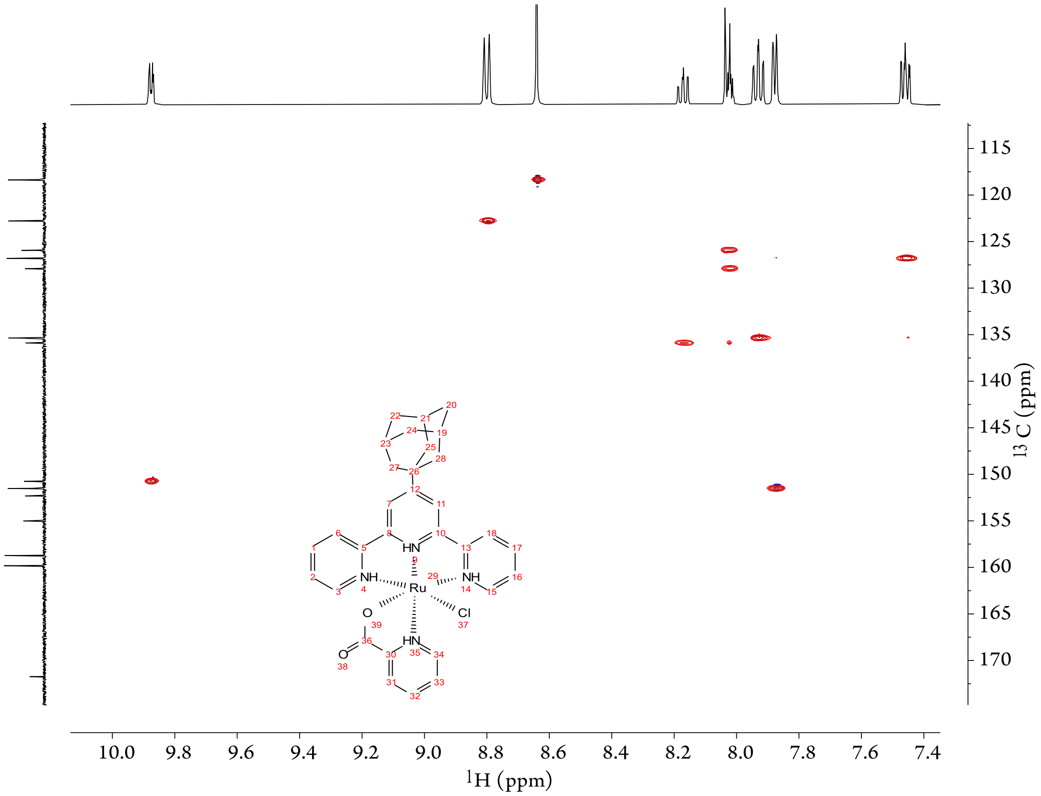

Figure S25. (a) Full spectrum b) zoomed in aromatic region HSQC (^{1}H – ^{13}C) NMR spectra of **2** in DMSO- d_6.

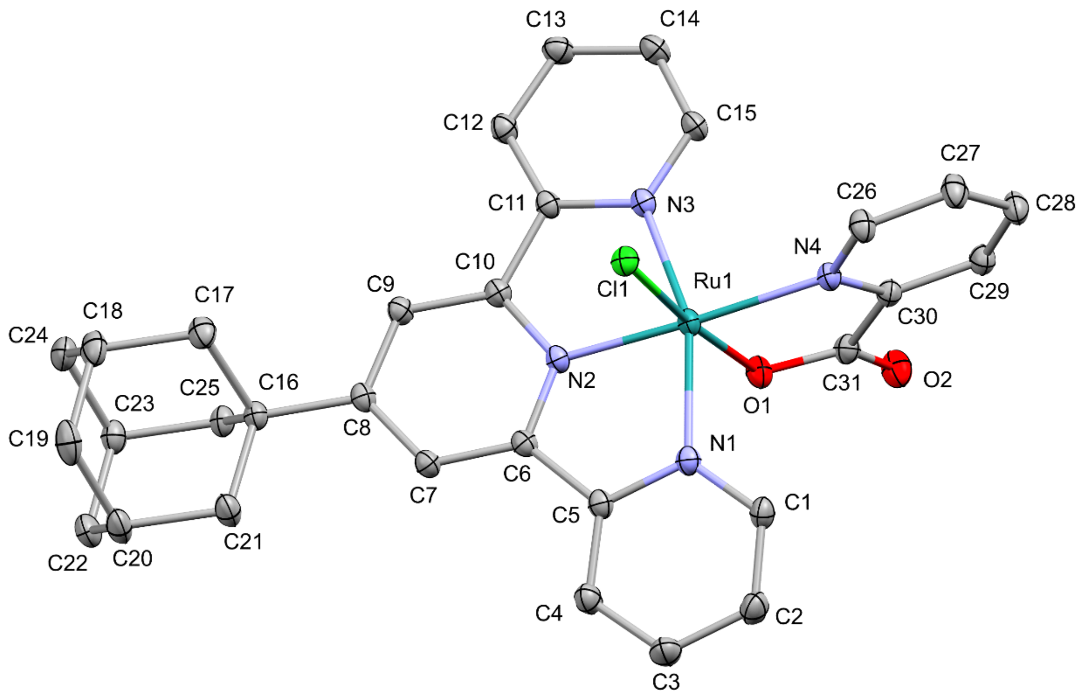

Figure S26. Crystal structure of **2**. Displacement ellipsoids are drawn at the 30% probability level. Solvent molecules and all H atoms were omitted for clarity.

The asymmetric unit contains two independent Ru molecules, one solvent molecule of DMSO (partially disordered), one solvent molecule of methanol (disordered over two sets of positions), and one molecule of water (disordered over two sets of positions).

Table S14. Crystallographic data for **2.**

Empirical formula	$C_{65}H_{70}Cl_2N_8O_7Ru_2S$
Formula weight	1380.39
Temperature/K	160.0(1)
Crystal system	triclinic
Space group	$P\bar{1}$
a/Å	13.85440(10)
b/Å	14.25840(10)
c/Å	17.11510(10)
α/°	84.4210(10)
β/°	82.4970(10)
γ/°	63.9560(10)
Volume/Å^3	3008.61(4)
Z	2
ρ_{calc}g/cm^3	1.524
μ/mm^{-1}	5.707
F(000)	1420.0
Crystal size/mm^3	0.14 × 0.03 × 0.01
Radiation	Cu Kα (λ = 1.54184)
2Θ range for data collection/°	5.214 to 159.502
Index ranges	-17 ≤ h ≤ 17, -17 ≤ k ≤ 18, -21 ≤ l ≤ 21
Reflections collected	65420
Independent reflections	12849 [R_{int} = 0.0268, R_{sigma} = 0.0202]
Data/restraints/parameters	12849/43/824
Goodness-of-fit on F^2	1.062
Final R indexes [I>=2σ (I)]	R_1 = 0.0261, wR_2 = 0.0642
Final R indexes [all data]	R_1 = 0.0283, wR_2 = 0.0653
Largest diff. peak/hole / e Å^{-3}	0.47/-0.78

Synthesis of 4-(1-Adamantyl) picolinic acid (ada-pic, **3b**)

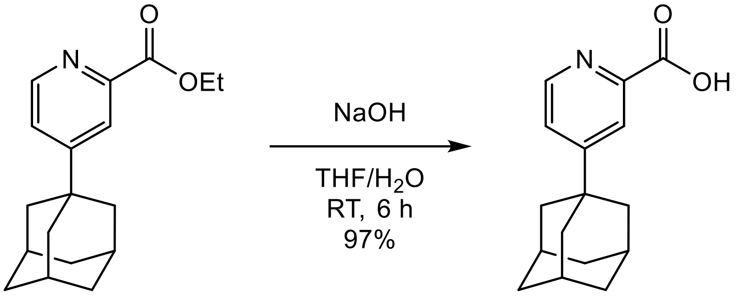

3a **3b**

Ethyl-4-(1-adamantyl)picolinate **3a**[47] (200 mg, 701 µmol) was dissolved in THF (6 mL) and aqueous NaOH (5 M, 2 mL) was added. The biphasic mixture was stirred at RT for 6 h before it was neutralised by the addition of aqueous HCl (5 M). The phases were separated, and the solvent of the organic phase evaporated to yield 4-(1-adamantyl)picolinic acid (**3b**, 175 mg, 680 µmol, 97%) as a yellow powder.

^{1}H NMR (400 MHz, CD_3OD, ppm): δ 8.85 (d, J = 6.1 Hz, 1H), 8.56 (d, J = 2.1 Hz, 1H), 8.33 (dd, J = 6.1, 2.1 Hz, 1H), 2.24–2.18 (m, 3H), 2.11–2.07 (m, 6H), 1.94–1.86 (m, 6H).

^{13}C NMR (100 MHz, CD_3OD, ppm): δ 174.8, 161.4, 143.6, 142.1, 128.2, 125.6, 42.6, 39.9, 37.1, 29.8.

HRMS (ESI) *m/z* calcd for $C_{16}H_{18}NO_2^-$ 256.1343 [M−H]$^-$; found: 256.1344.

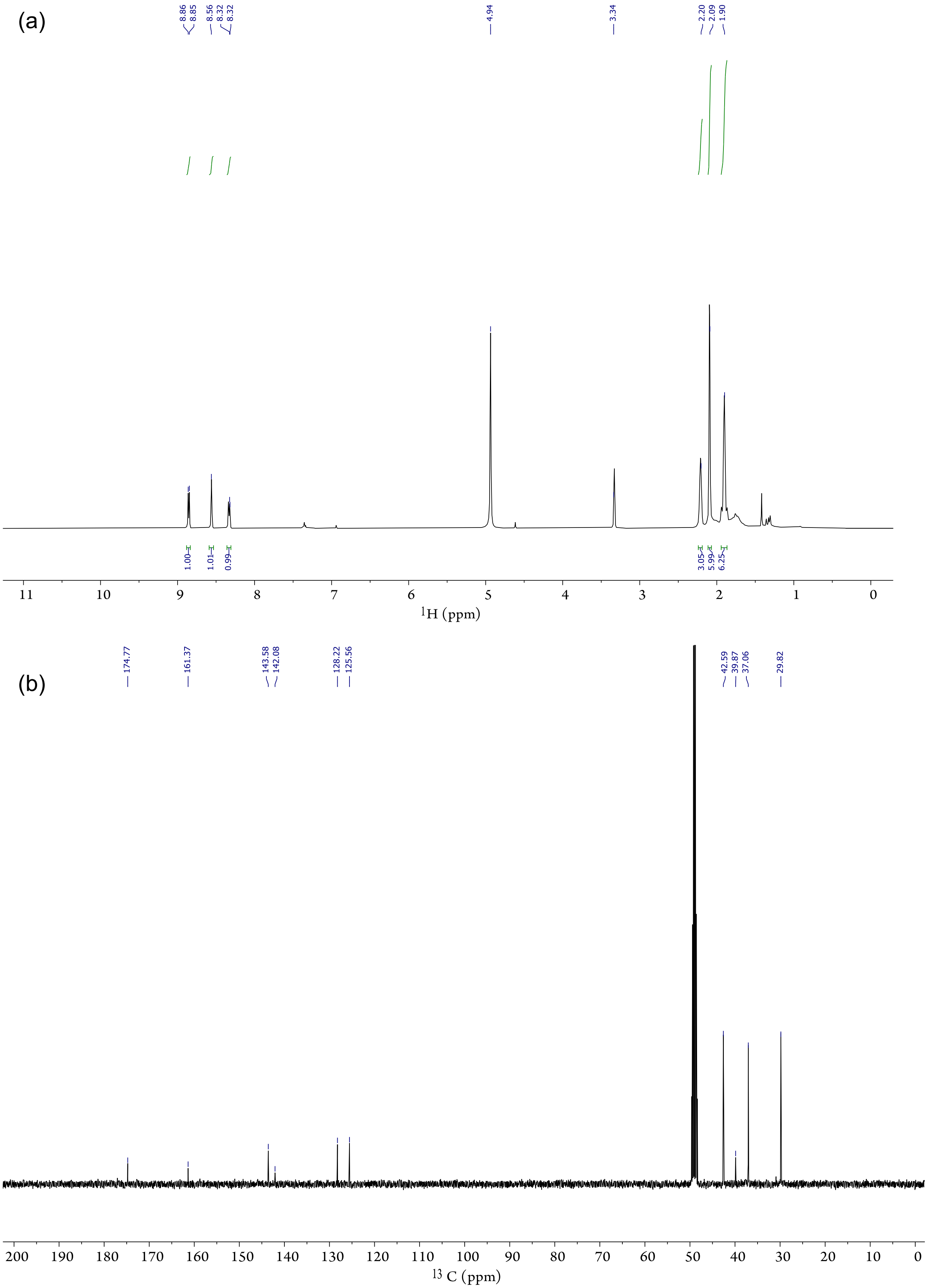

Figure S27. (a) ^{1}H and (b) ^{13}C NMR spectra of **3b** in CD_3OD.

Synthesis of [Ru(terpy)(ada-pic)(Cl)] 3

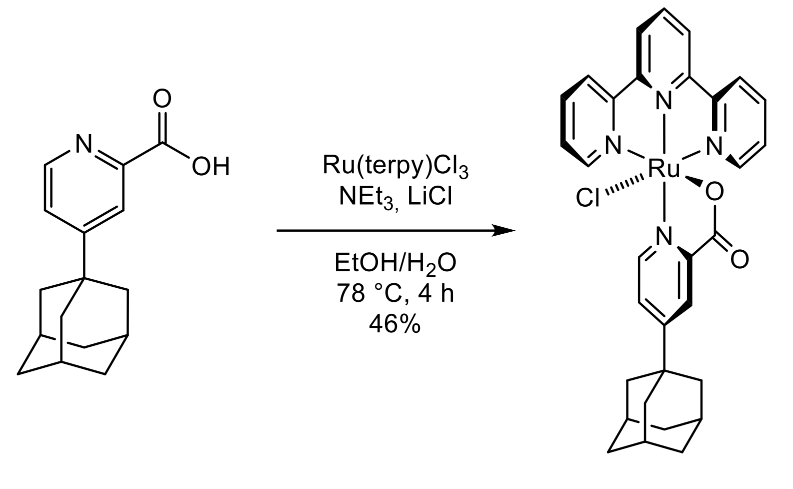

3b **3**

4-(1-Adamantyl)picolinic acid **3b** (120 mg, 466 µmol, 1 eq.) was dissolved in a mixture of EtOH/H_2O (1:1, 16 mL). LiCl (119 mg, 2.80 mmol, 6 eq.), NEt_3 (131 µL, 933 µmol, 2 eq.) and Ru(terpy)Cl_3 (206 mg, 466 µmol, 1 eq.) were added and the dark reaction mixture was heated to reflux for 4 h. The solvent was evaporated, and the crude product column chromatography (CH_2Cl_2/MeOH 20:1, R_f = 0.26) to yield [Ru(terpy)(ada-pic)Cl] (**3**, 134 mg, 214 µmol, 46%) as a dark purple powder.

^{1}H NMR (500 MHz, CD_3OD, ppm): δ 8.61 (d, J=5.2 Hz, 2H), 8.55 (d, J=8.0 Hz, 2H), 8.50 (d, J=8.0 Hz, 2H), 8.08 (d, J=2.2 Hz, 1H), 7.96 (td, J=7.8, 1.5 Hz, 2H), 7.91 (t, J=8.0 Hz, 1H), 7.64 (t, J=6.6 Hz, 2H), 7.02 (dd, J=6.1, 2.3 Hz, 1H), 6.77 (d, J=5.9 Hz, 2H), 2.05–2.01 (m, 3H), 1.81–1.69 (m, 12H).

^{13}C NMR (125 MHz, DMSO-d_6, ppm): δ 160.5, 158.9, 158.5, 155.4, 149.6, 135.9, 128.4, 127.22, 127.19, 124.12, 123.9, 122.6, 122.14, 121.9, 41.1, 35.9, 35.6, 27.8.

HRMS (ESI) *m/z* calcd for $C_{31}H_{30}ClN_4O_2Ru^+$: 627.10953 [M+H]$^+$; found: 627.10995

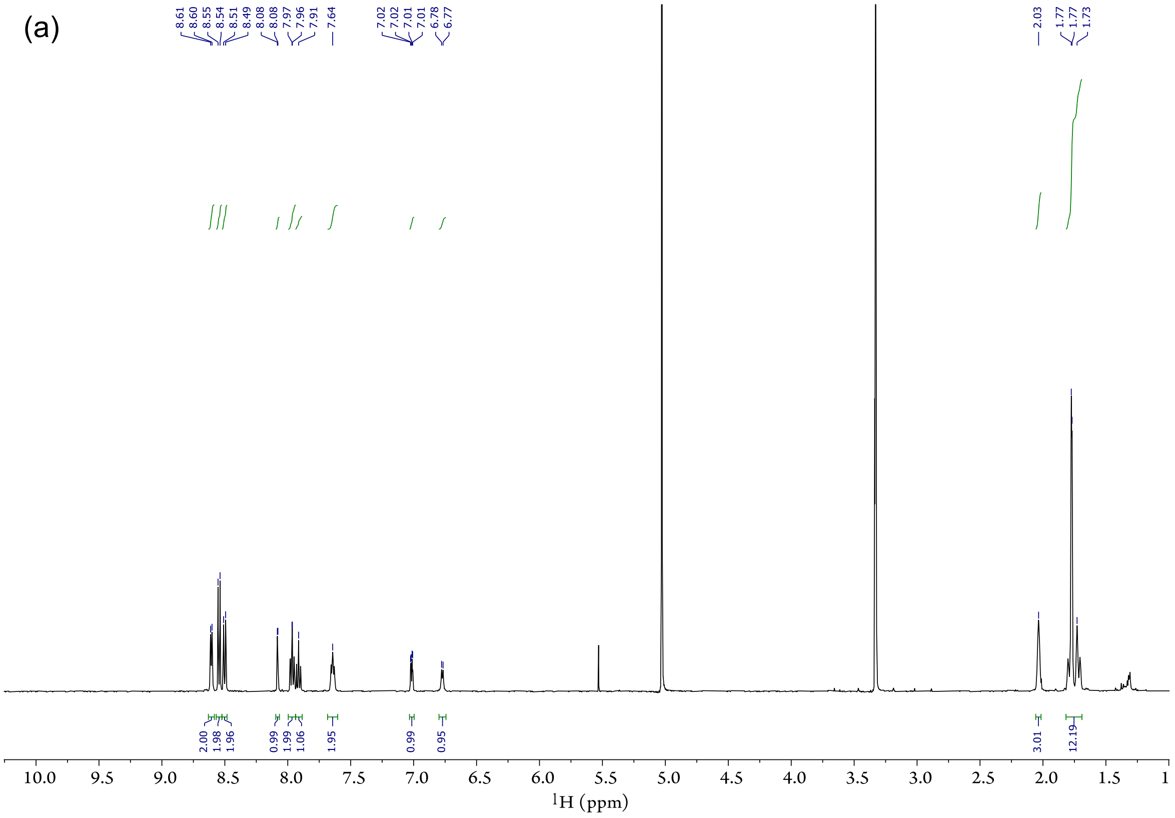

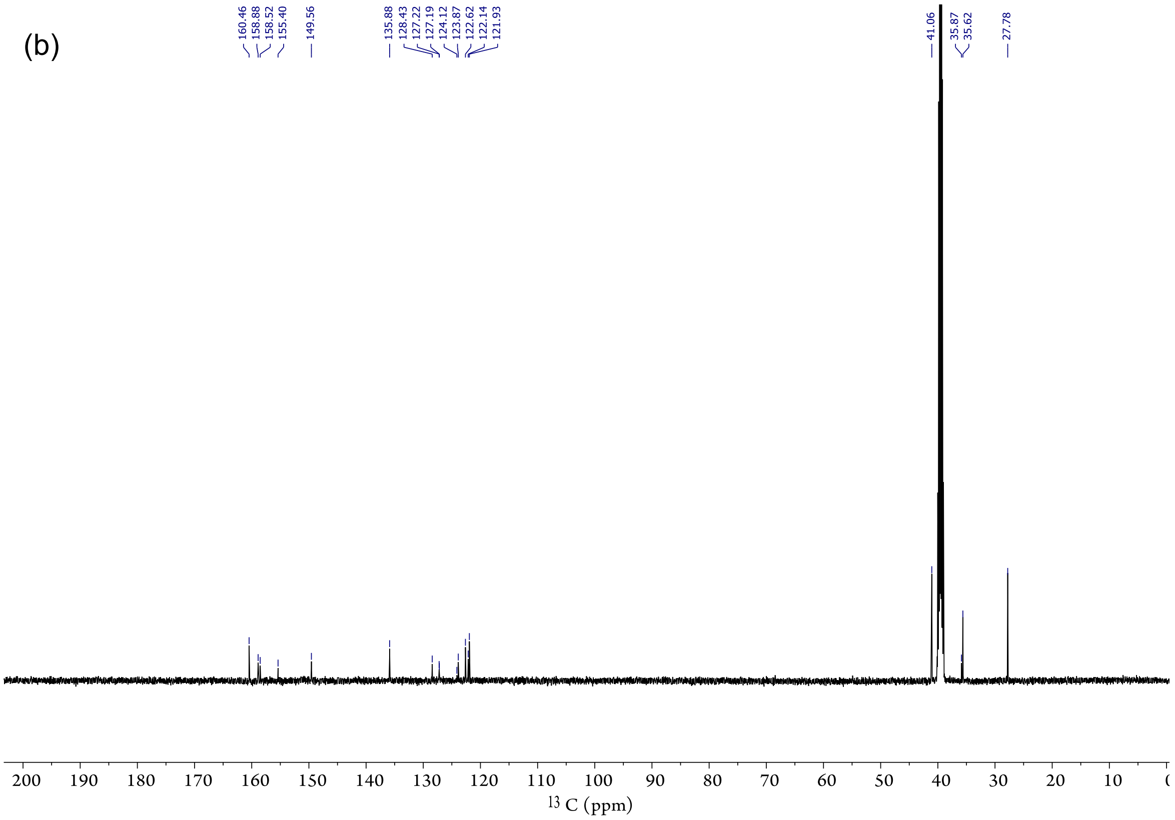

Figure S28. (a) ^{1}H NMR in CD_3OD and (b) ^{13}C NMR spectra in DMSO-d_6 of **3**.

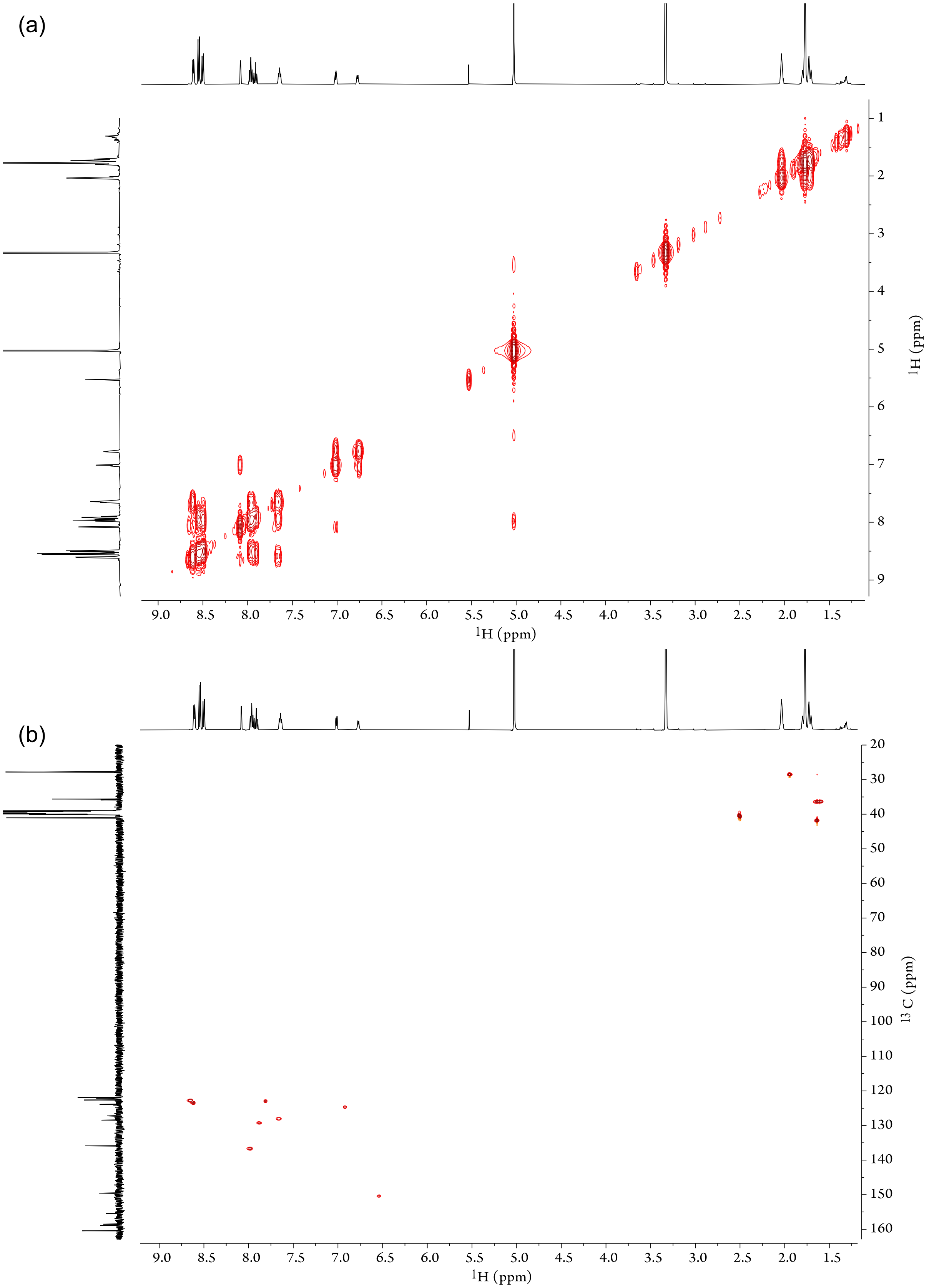

Figure S29. (a) HSQC (^{1}H – ^{13}C) and (b) COSY (^{1}H – ^{1}H) NMR spectra in DMSO-d_6.

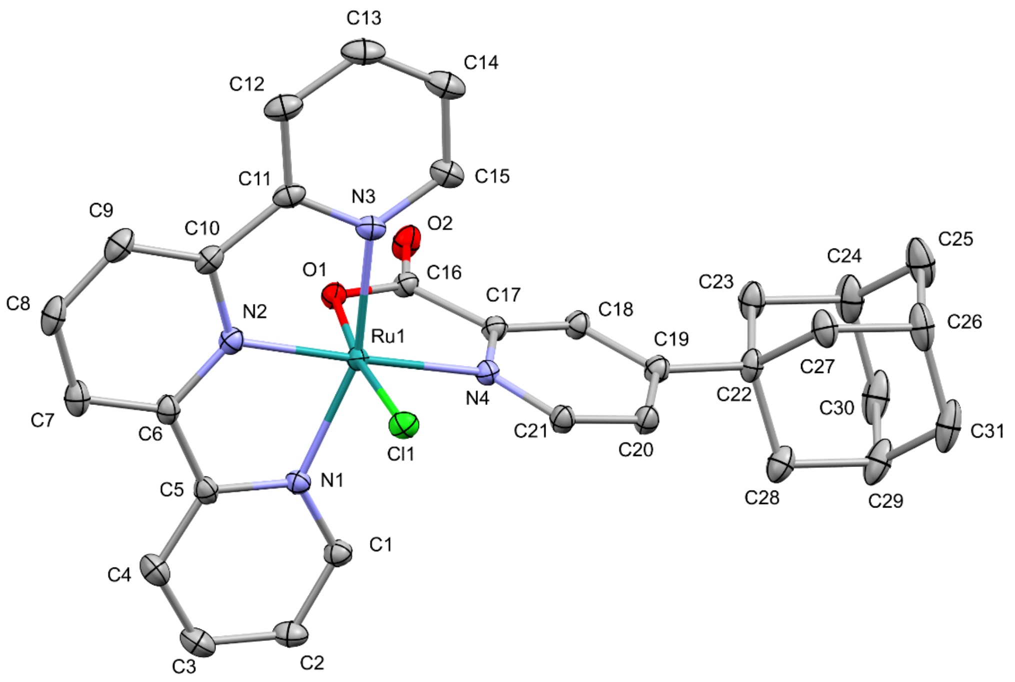

Figure S30. Crystal structure of **3**. Displacement ellipsoids are drawn at the 30% probability level. Solvent molecules and all H atoms were omitted for clarity.

Table S15. Crystallographic data of **3**.

Empirical formula	$C_{34}H_{41}ClN_4O_5Ru$
Formula weight	722.23
Temperature/K	160.0(1)
Crystal system	triclinic
Space group	$P\bar{1}$
a/Å	8.58170(10)
b/Å	13.7806(2)
c/Å	14.3462(3)
α/°	77.0170(10)
β/°	83.1010(10)
γ/°	83.6780(10)
Volume/Å^3	1635.18(5)
Z	2
ρ_{calc}g/cm^3	1.467
μ/mm^{-1}	5.019
F(000)	748.0
Crystal size/mm^3	0.14 × 0.08 × 0.01
Radiation	Cu Kα (λ = 1.54184)
2Θ range for data collection/°	6.354 to 159.818
Index ranges	-10 ≤ h ≤ 10, -17 ≤ k ≤ 12, -18 ≤ l ≤ 18
Reflections collected	54407
Independent reflections	7040 [R_{int} = 0.0237, R_{sigma} = 0.0144]
Data/restraints/parameters	7040/2/420
Goodness-of-fit on F^2	1.066
Final R indexes [I>=2σ (I)]	R_1 = 0.0265, wR_2 = 0.0693
Final R indexes [all data]	R_1 = 0.0270, wR_2 = 0.0696
Largest diff. peak/hole / e Å^{-3}	0.61/-0.69

Synthesis of EtO-Pillar[6]arene (Pa[6]OEt) (**4a**)

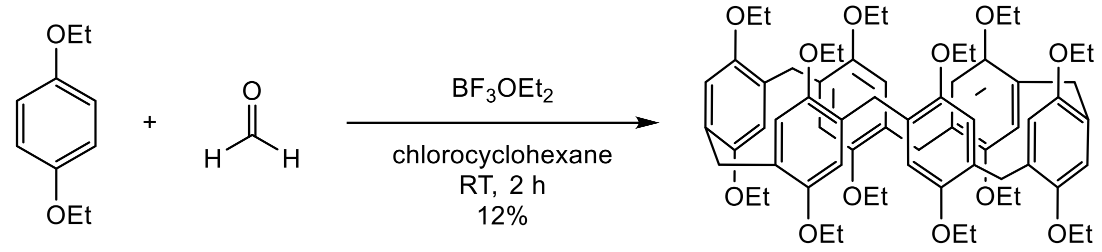

4a

1,4- Diethoxybenzene (1.2 g, 7.22 mmol) and paraformaldehyde (95%) (685 mg, 21.7 mmol, 3 equiv.) were suspended in 50 mL chlorocyclohexane[35] and 916 µL BF_3 OEt_2 (7.22 mmol) were added and the mixture was stirred under N_2 atmosphere, at RT (22 ºC) for 3 h. During this time, the reaction mixture turned from dark green to dark brown. Afterwards, a beaker with 200 mL fridge-cold MeOH was prepared, and the reaction mixture was added in small portions (pipette) to the cold MeOH under vigorous stirring. A white precipitate formed, which was filtered off, washed with MeOH and dried to give 936 mg (12%) Pillar[6]arene-OEt (**4a**).

^{1}H NMR (400 MHz, $CDCl_3$, ppm) δ 6.69 (s, 12H), 3.82 (m, *J* = 13.9, 6.9 Hz, 36H), 1.28 (t, *J* = 6.9 Hz, 36H).

^{13}C NMR (101 MHz, $CDCl_3$) δ 150.41 (Ar-**C**-O), 127.84 (Ar-**C**-CH2), 115.22 (Ar-C), 64.00 (O-**C**H_2), 30.93 (Ar-**C**H_2-Ar), 15.17 (**C**H_3).

HRMS (ESI$^+$): *m/z* calcd $C_{66}H_{84}O_{12}H^+$ 1069.6035 $[M+H]^+$; found 1069.6032; calcd $C_{66}H_{84}O_{12}Na^+$ 1091.5855 $[M+Na]^+$; 1091.5856; calcd $C_{66}H_{83}O_{12}HNH_4^+$ 1086.6301 $[M+ NH_4]^+$; found 1086.6304

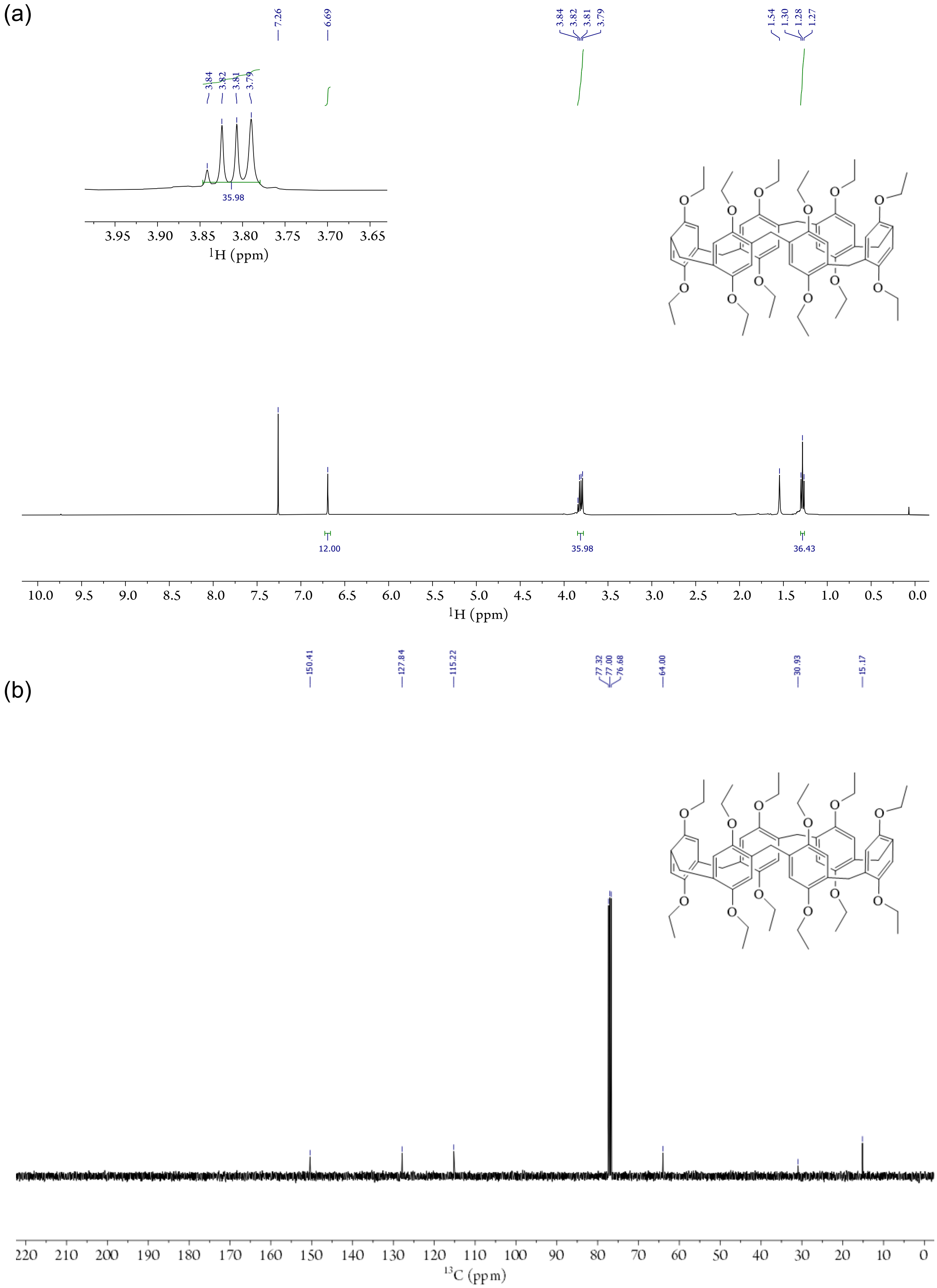

Figure S31. (a) ^{1}H NMR and (b) ^{13}C NMR spectra of **4a** in $CDCl_3$.

Synthesis of Pillar[6]arene-OH (PA[6], **4**)

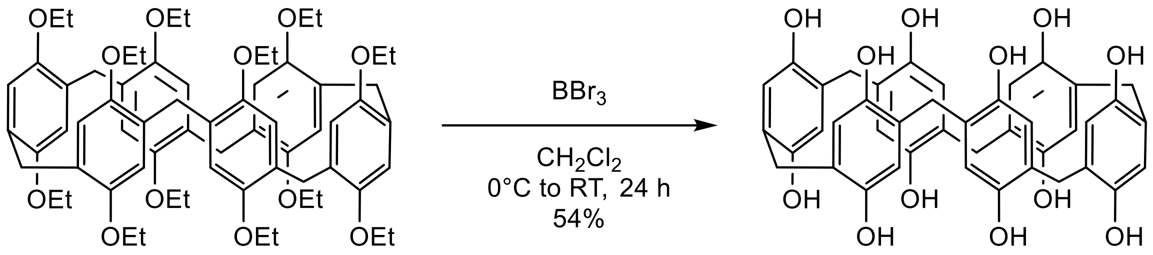

4a **4**

EtO-pillar[6]arene **4a** (700 mg, 0.655 mmol) were dissolved in dry CH_2Cl_2 with 3 Å molecular sieves. Afterwards, 26 equiv. (1.62 mL) BBr_3 were added at 0 °C and stirred for 1 h in an ice bath under N_2 atmosphere. The mixture was let warm to RT (22 °C) and stirred for 24 h. The reaction was quenched by adding H_2O to the mixture, forming a white-brown precipitate. The molecular sieves were removed by filtration with a strainer followed by filtering off the remaining solution with the precipitate. The cake was washed with HCl (pH 2), water and CH_2Cl_2. The resulting cake was dissolved in MeOH, filtered again, and the solvent of the filtrate was evaporated to give 260 mg (54%) pillar[6]arene (PA[6], **4**) as a beige brown powder. The dried compound is stored in the freezer.

^{1}H NMR (400 MHz, MeOD, ppm) δ 6.46 (s, 12H, aromatic-H), 3.65 (s, 12H, C**H₂**)

^{13}C NMR (101 MHz, MeOD, ppm) δ 148.42 (**C**-OH), 127.61 (Ar-**C**-CH_2), 118.24 (aromatic C-H), 30.64 (**C**H_2).

HRMS (ESI$^+$): *m/z* calcd $C_{42}H_{36}O_{12}Na^+$ 755.2099 [M+Na]$^+$; found 755.2098; calcd $C_{42}H_{36}O_{12}K^+$ 771.1838 [M+K]$^+$; found 771.1837; calcd $C_{42}H_{36}O_{12}NH_4^+$ 750.2545 [M+NH_4]$^+$ found 750.2545

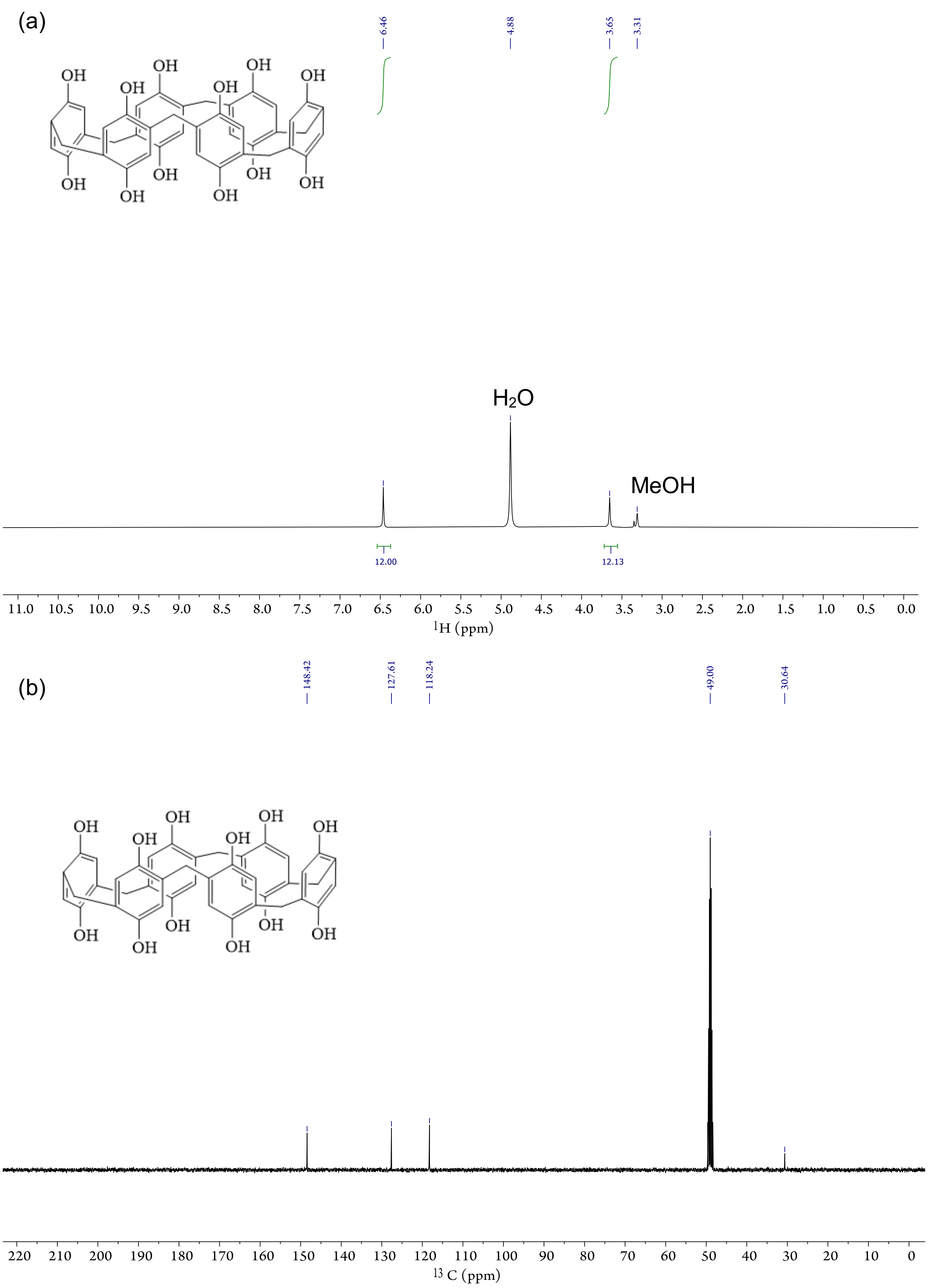

Figure S32. (a) ^{1}H NMR and (b) ^{13}C NMR spectra of **4** in MeOD.

References

[15] H. Roithmeyer, L. Sévery, T. Moehl, B. Spingler, O. Blacque, T. Fox, M. Iannuzzi, S. D. Tilley, *J. Am. Chem. Soc.* **2024**, *146*, 430–436.

[19] J. Bühler, A. Muntwyler, H. Roithmeyer, P. Adams, M. L. Besmer, O. Blacque, S. D. Tilley, *Chem. Eur. J.* **2024**, *30*, e202304181.

[35] T. Ogoshi, N. Ueshima, T. Akutsu, D. Yamafuji, T. Furuta, F. Sakakibara, T. Yamagishi, *Chem. Commun.* **2014**, *50*, 5774–5777.

[40] A. J. Bard, L. R. Faulkner, *Electrochemical Methods: Fundamentals and Applications, 2nd Edition*, John Wiley & Sons, Inc., New York, **2001**.

[47] B. P. Sullivan, J. M. Calvert, T. J. Meyer, *Inorg. Chem.* **1980**, *19*, 1404–1407.

[48] T. Sasaki, K. Shimizu, M. Ohno, *Chem. Pharm. Bull.* **1984**, *32*, 1433–1440.

[49] H. E. Gottlieb, V. Kotlyar, A. Nudelman, *J. Org. Chem.* **1997**, *62*, 7512–7515.

[50] R. C. Clark, J. S. Reid, *Acta Cryst. A* **1995**, *51*, 887–897.

[51] CrysAlisPro (Version 1.171.43.104a), Rigaku Oxford Diffraction Ltd, Yarnton, Oxfordshire, England, **2022**.

[52] O. V. Dolomanov, L. J. Bourhis, R. J. Gildea, J. A. K. Howard, H. Puschmann, *J. Appl. Cryst.* **2009**, *42*, 339–341.

[53] G. M. Sheldrick, *Acta Cryst. A* **2015**, *71*, 3–8.

[54] G. M. Sheldrick, *Acta Cryst. C* **2015**, *71*, 3–8.

[55] A. L. Spek, *Acta Cryst. D* **2009**, *65*, 148–155.

[56] AAT Bioquest, Inc., "Quest Calculate™ Potassium Phosphate (pH 5.8 to 8.0) Preparation and Recipe", can be found under https://www.aatbio.com/resources/buffer-preparations-and-recipes/potassium-phosphate-ph-5-8-to-8-0, **2024** (accessed 22.05.24).

[57] T. D. Kühne, M. Iannuzzi, M. Del Ben, V. V. Rybkin, P. Seewald, F. Stein, T. Laino, R. Z. Khaliullin, O. Schütt, F. Schiffmann, D. Golze, J. Wilhelm, S. Chulkov, M. H. Bani-Hashemian, V. Weber, U. Borštnik, M. Taillefumier, A. S. Jakobovits, A. Lazzaro, H. Pabst, T. Müller, R. Schade, M. Guidon, S. Andermatt, N. Holmberg, G. K. Schenter, A. Hehn, A. Bussy, F. Belleflamme, G. Tabacchi, A. Glöß, M. Lass, I. Bethune, C. J. Mundy, C. Plessl, M. Watkins, J. VandeVondele, M. Krack, J. Hutter, *J. Chem. Phys.* **2020**, *152*, 194103.

[58] J. P. Perdew, K. Burke, M. Ernzerhof, *Phys. Rev. Lett.* **1996**, *77*, 3865–3868.

[59] R. Sabatini, T. Gorni, S. de Gironcoli, *Phys. Rev. B* **2013**, *87*, 041108.

[60] J. VandeVondele, J. Hutter, *J. Chem. Phys.* **2007**, *127*, 114105.

[61] S. Goedecker, M. Teter, J. Hutter, *Phys. Rev. B.* **1996**, *54*, 1703–1710.

[62] Materials Project, "In2O3; database version v2023.11.1", can be found under https://next-gen.materialsproject.org/materials/mp-22598, **2023** (accessed 22.05.24).

[63] A. Hjorth Larsen, J. Jørgen Mortensen, J. Blomqvist, I. E. Castelli, R. Christensen, M. Dułak, J. Friis, M. N. Groves, B. Hammer, C. Hargus, E. D. Hermes, P. C. Jennings, P. Bjerre Jensen, J. Kermode, J. R. Kitchin, E. Leonhard Kolsbjerg, J. Kubal, K. Kaasbjerg, S. Lysgaard, J. Bergmann Maronsson, T. Maxson, T. Olsen, L. Pastewka, A. Peterson, C. Rostgaard, J. Schiøtz, O. Schütt, M. Strange, K. S. Thygesen, T. Vegge, L. Vilhelmsen, M. Walter, Z. Zeng, K. W. Jacobsen, *J. Phys.: Condens. Matter* **2017**, *29*, 273002.

[64] P. Thordarson, "BindFit v0.5", can be found under http://app.supramolecular.org/bindfit/, **2024** (accessed 22.05.24).
